# Supplementary material for: Real-time single-cell characterization of the eukaryotic transcription cycle reveals correlations between RNA initiation, elongation, and cleavage
Source: PLoS Comput Biol. 2021 May 18;17(5):e1008999. doi: 10.1371/journal.pcbi.1008999 (PMC8162642; doi:10.1371/journal.pcbi.1008999)
Supplement: S1 File — Table A: Mean and standard deviation of model parameters used in single-cell simulations. Table B: Comparison of Spearman rank correlation coefficients and p-values between experimental and simulated single-cell correlations. Table C: Parameters used in single-molecule Monte Carlo simulation of elongation rates. (PDF) [file pcbi.1008999.s001.pdf]

Fig S1. Detailed description of reporter construct used in this work. Labeled positions are $x^{start}_{MS2} = 0.024$ kb, $x^{end}_{MS2} = 1.299$ kb, $x^{start}_{PP7} = 4.292$ kb, and $x^{end}_{PP7} = 5.758$ kb, where $x = 0$ corresponds to the 3' end of the promoter. Distances are $d = 4.27$ kb and $L = 6.63$ kb.

S1 Supplementary Information

A Full Model

To predict MS2 and PP7 fluorescence traces, we utilized a simple model of transcription initiation, elongation, and cleavage. The entire model has the following free parameters:

- $\langle R \rangle$, the mean transcription initiation rate
- $\delta R(t)$, the time-dependent fluctuations in the transcription initiation rate around the mean $\langle R \rangle$
- v_{elon}, the RNAP elongation rate
- τ_{cleave}, the mRNA cleavage time
- t_{on}, the time of transcription onset after the previous mitosis, where $t = 0$ corresponds to the start of anaphase
- MS2_{basal}, the basal level of MCP-mCherry fluorescence
- PP7_{basal}, the basal level of PCP-eGFP fluorescence
- α, the scaling factor between MCP-mCherry and PCP-eGFP arbitrary fluorescence units

Note that the fluctuations $\delta R(t)$ are independent for each time point, and exist to allow for a slight time dependence in the overall initiation rate. Thus, $\delta R(t)$ parameterizes a set of independent constant offsets in the overall loading rate at each time point.

First, the parameters $\langle R \rangle$, $\delta R(t)$, t_{on}, v_{elon}, and τ_{cleave} were used to generate a map $x_i(t)$ of the position of each actively transcribing RNAP molecule i along the body of the reporter gene, as a function of time. Although the model is represented with continuous time, the subsequent computational simulation used for the statistical inference relies on discrete timesteps. Thus, given a computational time step dt, $R(t)dt$ RNAP molecules are loaded at time point t at the promoter $x = 0$, where

$$R(t) = \begin{cases} 0 & t < t_{on} \\ \langle R \rangle + \delta R(t) & t \geq t_{on}. \end{cases}$$

Note while $R(t)dt$ is a floating point number, the model utilizes discrete numbers of RNAP molecules. As a result, $R(t)dt$ is rounded down to the nearest integer since the model cannot load fractional numbers of RNAP molecules. After initiation, each RNAP molecule proceeds forward with the constant elongation rate v_{elon}. Once an RNAP molecule reaches the end of the gene, an additional cleavage time τ_{cleave} elapses after which the nascent transcript is cleaved and disappears instantly. This assumption of instantaneous disappearance following cleavage is justified in Section C in S1 File based on the diffusion time scale of individual mRNA molecules.

From this position map, and based on the locations of the stem loop sequences along the reporter construct (S1 Fig), we calculate the predicted MS2 and PP7 fluorescence

signals. The contribution to the MS2 signal $F_i^{MS2}(t)$ of an individual RNAP molecule i at position $x_i(t)$ is given by

$$F_i^{MS2}(t) = \begin{cases} 0 & x_i(t) < x_{MS2}^{start} \\ \frac{x_i(t) - x_{MS2}^{start}}{x_{MS2}^{end} - x_{MS2}^{start}} F_{MS2} & x_{MS2}^{start} \leq x_i(t) < x_{MS2}^{end} \\ F_{MS2} & x_i(t) \geq x_{MS2}^{end} \end{cases},$$

where x_{MS2}^{start} and x_{MS2}^{end} are the start and end positions of the MS2 stem loop sequence, respectively, and F_{MS2} is the mCherry fluorescence produced by a single RNAP molecule that has transcribed the entire set of MS2 stem loops. Here, we also assume that RNAP molecules that have only partially transcribed the MS2 stem loops result in a fractional fluorescence given by the fractional length of the MS2 stem loop sequence transcribed. Similarly, the contribution to the PP7 signal $F_i^{PP7}(t)$ is given by

$$F_i^{PP7}(t) = \begin{cases} 0 & x_i(t) < x_{PP7}^{start} \\ \frac{x_i(t) - x_{PP7}^{start}}{x_{PP7}^{end} - x_{PP7}^{start}} F_{PP7} & x_{PP7}^{start} \leq x_i(t) < x_{PP7}^{end} \\ F_{PP7} & x_i(t) \geq x_{PP7}^{end} \end{cases},$$

where x_{PP7}^{start} and x_{PP7}^{end} are the start and end positions of the PP7 stem loop sequence, respectively, and F_{PP7} is the GFP fluorescence produced by a single RNAP molecule that has transcribed the entire set of PP7 stem loops. Note that we assume that the MCP-mCherry and PCP-GFP fluorophores effectively bind instantaneously to all their associated stem loops once they are transcribed. Due to the high numbers of nascent transcripts on the reporter gene (Fig 5D), we expect that corrections to this assumption due to incomplete, stochastic, and/or non-instantaneous fluorophore binding will not introduce substantial deviations to the model.

The temporal dynamics of the total MS2 and PP7 signals $F_{MS2}(t)$ and $F_{PP7}(t)$ are then obtained by summing over all the individual RNAP molecule contributions for each timepoint

$$F_{MS2}(t) = \sum_{i=1}^{N} F_i^{MS2}(t)$$
$$F_{PP7}(t) = \sum_{i=1}^{N} F_i^{PP7}(t),$$

where i is the index of each individual RNAP molecule and N is the total number of loaded RNAP molecules. The final signal is then modified by accounting for the scaling factor α and the basal fluorescence values of MS2_{basal} and PP7_{basal}. α is necessary because the two fluorescent protein signals have different arbitrary units (Fig 3). Further, the two basal fluorescence values are incorporated to account for the experimentally observed low baseline fluorescence in each fluorescent channel. The final signals $F'_{MS2}(t)$ and $F'_{PP7}(t)$ are then given by

$$F'_{MS2}(t) = \begin{cases} \text{MS2}_{basal}/\alpha & F_{MS2}(t) < \text{MS2}_{basal} \\ F_{MS2}(t)/\alpha & F_{MS2}(t) \geq \text{MS2}_{basal} \end{cases}$$

and

$$F'_{PP7}(t) = \begin{cases} \text{PP7}_{basal} & F_{PP7}(t) < \text{PP7}_{basal} \\ F_{PP7}(t) & F_{PP7}(t) \geq \text{PP7}_{basal}. \end{cases}$$

All of the model parameters introduced in this section were used as free parameters in the fitting procedure described in Section D in S1 File.

Note that the model does not make mechanistic claims about the nature of the cleavage process, which could potentially be convolved with processes such as transcriptional pausing. Specifically, if RNAP pausing were to happen 3' of the PP7 stem loop sequence, then it is effectively indistinguishable from cleavage at the 3' UTR.

However, we stress that our model is only an effective parameterization, and so we make no mechanistic claims as to the source of a particular cleavage time value. What our model interprets as cleavage could stem from pausing at the 3'UTR of the reporter, for example, or from continued elongation past the 3'UTR due to inefficient cleavage and termination processes. These would exhibit the same experimental signals—namely, persistence of fluorescent signal after the expected time of signal loss—and thus is a challenge of experimental resolution and not of model formulation.

B Characterization of photobleaching in experimental setup

To determine whether photobleaching was present in our experimental setup, we conducted an experiment with the dual-color 5'/3' tagged reporter (Fig 1C) where half of the field of view was illuminated using the experimental settings described in the Methods and Materials section (S2A Fig, purple), and the other half was illuminated at half the temporal sampling rate (S2A Fig, yellow).

Since the measurement conditions were identical except for the sampling rate for both reporter constructs used in this work, any systematic differences between the two measurement conditions could only stem from this different sampling rate. Thus, if the experimental settings were in the photobleaching regime, then the purple region would exhibit fluorescence at a systematically lower intensity compared to the yellow region. S2B Fig and S2C Fig show the fluorescence intensities of mCherry and eGFP as a function of time at a particular anterior-posterior position of the embryo for both 0.5x and 1x sampling rates, where data points indicate fluorescence averaged within the anterior-posterior position (indicated schematically by the dashed box in S2A Fig) and error bars indicate standard error across cells. The plots reveal that, qualitatively, there is no obvious systematic difference between the two illumination regions.

To quantify photobleaching, we defined the average normalized difference Δ between illuminated regions. This magnitude is calculated by subtracting the fluorescence value at 1x sampling rate F_{1x} by that at 0.5x sampling rate, dividing by the fluorescence value at 0.5x sampling rate $F_{0.5x}$, and then averaging across all time points $N_{timepoints}$ and embryo positions $N_{positions}$

$$\Delta = \sum_{i=1}^{N_{timepoints}} \sum_{j=1}^{N_{positions}} \frac{1}{N_{timepoints}} \frac{1}{N_{positions}} \frac{F_{1x}^{ij} - F_{0.5x}^{ij}}{F_{0.5x}^{ij}}.$$

For example, for the curves shown in S2B Fig, this entails subtracting the red curve by the black curve, dividing by the black curve, and then averaging for all anterior-posterior embryo positions. An overall value of less than zero means that the 1x sampling rate produces systematically lower fluorescence intensities, indicating that our experimental settings are in the photobleaching regime.

As seen in S2D Fig, the average normalized difference Δ is consistent with zero for both fluorophores (within standard error, measured across all time points and anterior-posterior positions). Thus, we conclude that our data are not in the photobleaching regime.

Fig S2. Investigation of photobleaching in experimental setup. (A) Control experiment where half of the field of view is illuminated at the standard experimental settings (yellow), and the other half of the field of view is imaged at half of the illumination rate (purple). (B, C) The (B) mCherry and (C) eGFP fluorescence signals at a given anterior-posterior embryo position, averaged across cells within that position (white dashed rectangle in (A)), do not exhibit photobleaching. (D) The average normalized difference between illuminated regions, averaged across time points and anterior-posterior embryo positions, are approximately zero within error. A negative value would indicate the presence of photobleaching. (B, C, error bars indicate standard error of the mean averaged across cell nuclei in the field of view; D, error bars indicate standard error of the mean averaged across time points and embryo positions).

C Justification for approximating transcript cleavage as instantaneous

In the model presented in Section A in S1 File, we assumed that, when a nascent RNA transcript is cleaved at the end of the reporter gene, its MS2 and PP7 fluorescence signals disappear instantaneously. Here, we justify this assumption by demonstrating that the timescale of mRNA diffusion away from the active locus is much shorter than the experimental resolution of our system.

When a nascent RNA transcript is cleaved, it diffuses away from the gene locus. For a free particle with diffusion coefficient D, the characteristic timescale τ to diffuse a length scale L is given by

$$\tau \sim \frac{L^2}{D}.$$

In the context of the experiment performed here, this can interpreted as the timescale for a cleaved mRNA transcript to diffuse away from the diffraction-limited fluorescence punctum at the locus.

We can estimate the characteristic timescale τ by plugging in the following values. Assume that the completed transcript possesses a typical mRNA diffusion coefficient of $D \sim 0.1\ \mu m^2/s$ [149]. The length scale L corresponds to the Abbe diffraction limit, which yields $L \sim 250\ nm$ for green light with a wavelength of about 500 nm and a microscope with a numerical aperture of 1. Plugging these values into the equation yields a diffusion time scale of

$$\tau \sim \frac{(250nm)^2}{0.1\mu m^2/s} \sim 0.625\ s.$$

As a result, a newly cleaved mRNA transcript will typically diffuse away from the locus in less than a second, meaning that its MS2 and PP7 fluorescence signal will vanish much faster than our experimental time resolution of 15 s. For this reason, we can justify approximating the cleavage process as instantaneously removing the fluorescent signals of newly cleaved transcripts.

D Overview and application of MCMC

The inference procedures described in the main text were carried out using the established technique of Markov Chain Monte Carlo (MCMC). Specifically, we used the MATLAB package MCMCstat, an adaptive MCMC technique [146,147]. For detailed descriptions, we refer the reader to the the MCMCstat website (`https://mjlaine.github.io/mcmcstat/`), as well as to a technical overview of

MCMC [69]. Briefly, MCMC allows for an estimation of the parameter values of a model that best fit the experimentally observed data along with an associated error. In this work, we use MCMC to infer the best fit values of the transcription cycle parameters given observed fluorescence data at the single-cell level. Then, we combine these inference results across cells to construct distributions of inferred values across the ensemble of cells.

MCMC calculates a Bayesian posterior probability distribution of each free parameter given the data by stochastically sampling different parameter values. For a given set of observations D and a model with parameters θ, the so-called posterior probability distribution of θ possessing a particular set of values is given by Bayes' theorem

$$\underbrace{p(\theta|D)}_{\text{posterior}} = \frac{\overbrace{p(D|\theta)}^{\text{likelihood}}\ \overbrace{p(\theta)}^{\text{prior}}}{\underbrace{p(D)}_{\text{evidence}}}.$$

This posterior distribution is a combination of three components: the likelihood, prior, and evidence. This latter term represents the probability of the observations possessing their particular values, and allows the overall posterior distribution to be normalized. In practice, the evidence term is often dropped since MCMC can still yield accurate results without requiring this normalization. Thus, we have

$$\underbrace{p(\theta|D)}_{\text{posterior}} \propto \overbrace{p(D|\theta)}^{\text{likelihood}}\ \overbrace{p(\theta)}^{\text{prior}}.$$

The prior function contains *a priori* assumptions about the probability distribution of parameter values θ, and the likelihood function represents the probability of obtaining the observations, given a particular set of parameters θ. Thus, the *most likely* set of parameters θ occurs when the product of the likelihood and prior is maximized, resulting in a maximum in the posterior function. MCMC extends this by sampling different values of θ such that an approximation of the full posterior distribution is also obtained.

The prior distributions for the inferred parameters were set as follows. The prior distribution for the fluctuations in the initiation rate $\delta R(t)$ at each time point was assumed to be a Gaussian distribution centered around 0 AU/min with a standard deviation of 30 AU/min. This penalized fluctuations that strayed too far from zero, smoothing the overall initiation rate $R(t)$. For the rest of the parameters, a uniform distribution was chosen using the following uniform intervals:

- v_{elon}: [0, 10] kb/min
- t_{on}: [0, 10] min
- α: [0, 1]
- τ_{cleave}: [0, 20] min
- $MS2_{basal}$: [0, 50] AU
- $PP7_{basal}$: [0, 50] AU
- $\langle R \rangle$: [0, 40] AU/min

These intervals were justified with the following arguments. Previous elongation rate measurements have indicated values between around 1 and 4 kb/min (S9 Fig; [89]), so

we approximately doubled this range for flexibility. Previous measurements of the transcription onset time t_{on} for *hunchback* range from about 1 to 6 min [25], so we chose a similarly flexible interval. The calibration factor α must take on values between 0 and 1, since, under the experimental settings used, mCherry exhibits weaker absolute fluorescence than eGFP (see for example, Fig 3C). Although the cleavage time is not well understood, estimates lie on the order of minutes [75]—we chose a large interval to be conservative. Based on our experimental data (e.g. Fig 2B), basal levels of MS2 and PP7 fluorescence lie comfortably in the range [0, 50] AU. Finally, as observed in our data and also reported in [25], the mean rates of initiation lie comfortably in the range [0, 40] AU/min (Fig 4A).

For the likelihood function, a Gaussian error function was used

$$p(D|\theta) = e^{-SS},$$

where SS is a scaled sum-of-squares residual function given by

$$SS = \sum_t \frac{(F_{data} - F_{prediction})^2}{F_{data}}. \tag{A}$$

Here, the summation runs over individual time points, F_{data} corresponds to the MS2 or PP7 fluorescence at a given timepoint, and $F_{prediction}$ corresponds to the predicted MS2 or PP7 fluorescence according to the model, for a given set of parameter values. That is,

$$F_{data} = \left\{\mathrm{MS2}_1, \ldots, \mathrm{MS2}_N, \mathrm{PP7}_1, \ldots \mathrm{PP7}_N\right\}$$

where the subscripts indicate the time index over N time points. Similarly,

$$F_{prediction} = \left\{\mathrm{MS2}_1^{pred}, \ldots, \mathrm{MS2}_N^{pred}, \mathrm{PP7}_1^{pred}, \ldots \mathrm{PP7}_N^{pred}\right\}$$

where the superscripts indicate that these are model predictions evaluated at the experimental time points. The presence of F_{data} in the denominator scales the overall sum-of-squares residual function by the mean signal intensity and is required because the measurement noise in the fluorescence scales linearly with fluorescence intensity (Section E in S1 File and S3 Fig).

The MCMC approach samples values of parameters θ to approximate the posterior probability distribution. There are several algorithms that achieve this—the adaptive technique used in the MCMCstat package is an efficient algorithm that updates the sampling technique to more quickly arrive at the converged distribution.

For each inference run, an initial condition of parameter values is chosen. The algorithm then stochastically updates the next set of parameter values based on the current and previous values of the posterior distribution function. After a preset number of updates (typically at least on the order of thousands), the algorithm stops, resulting in a *chain* of MCMC parameter value samples. The initial period following the initial condition, known as the *burn-in* time, is typically discarded since the results are not reliable. The remaining values of the chain comprise an approximation of the underlying posterior probability distribution, with smaller errors for longer run times.

For the purposes of this work, the MCMC procedure was run by separately inferring parameter values for the data corresponding to each single cell. For each inference, random parameter values were chosen for the initial condition of the sampling algorithm in order to prevent initial condition bias from affecting the inference results. The algorithm was run for a total of $20,000$ iterations, which, after removing a burn-in window of length $10,000$, resulted in a chain of length $10,000$ for each of the 355 cells examined. To assess whether or not the algorithm was run for a sufficient number of iterations, the final chain was examined for *rapid mixing*, where the sampled values of a

particular parameter rapidly fluctuate around a converged value. Fig 2C highlights this rapid mixing in the inferred transcription cycle parameters of a sample single cell. The lack of long-timescale correlations, also exemplified by the quick decay of the auto-correlation function of each chain (Fig 2D), indicates that the algorithm has converged. In addition, a corner plot of the three transcription cycle parameters (Fig 2E) illustrates the pairwise correlations between them, demonstrating that the inference did not encounter degenerate solutions, and that each parameter has a fairly unimodal distribution.

These diagnostics provided a check on the quality of the inference results. Afterwards, the mean value of each parameter's final chain was then retained for each single cell for use in the further statistical analysis carried out in the main text.

E Justification of scaled observation model due to fluorescence noise behavior

The observation model parameterized by the sum-of-squares residual in Equation A in S1 File is scaled by dividing by the overall fluorescence intensity. This is needed because the fluorescence noise is not constant, but rather scales linearly with overall intensity. Here, we demonstrate this behavior by examining the fluorescence noise exhibited in our system.

A priori, if we consider that the fluorescent signals in our experiment are the result of the sum of many individual fluorophores, then we would expect that, if an individual fluorophore possesses some intrinsic constant measurement error with variance σ^2, then the associated error of N fluorophores would have a similarly scaled overall measurement error with variance $N\sigma^2$. Since N is proportional to the overall mean fluorescent signal, the observation model in Equation A in S1 File thus needs the mean signal in the denominator.

To validate this scaling of the variance with the mean, we examined the data from the dual-color interlaced MS2/PP7 reporter construct from Fig 3B. These data constitute, in principle, a two-point measurement of the same underlying biological process, so we reasoned that we could utilize this measurement to quantify the scaling of fluorescence noise with respect to overall fluorescence intensity.

Specifically, by creating bins of eGFP fluorescence measurement from the scatterplot in Fig 3D, we calculated how the variance of associated mCherry fluorescence values within a bin scaled with eGFP fluorescence (here a proxy for overall fluorescence intensity). If the calculated variance increased with overall fluorescence, this would indicate that the fluorescence measurement noise is not constant, but rather scaled positively with signal strength. S3 Fig shows this calculated variance (red), along with bootstrapped standard error, as a function of bin value (i.e. eGFP fluorescence). We see that the variance indeed increases with bin value fairly linearly, confirming our hypothesis. If we then scale the variances by dividing by the mean mCherry fluorescence within a bin, we recover a constant scaling, as expected (black).

The fluorescence intensity of each detected MS2 or PP7 spot was calculated by integrating the pixel intensities in a small circular neighborhood with a fixed radius of about 1 micron around each spot center and subtracting by the background fluorescence, calculated by fitting a Gaussian to the spatial fluorescence profile (see Methods and Materials). While the number of detected pixels does contributes to the fluorescence intensity (and thus variance across measurements), the size of a spot does correlate with overall transcriptional activity – thus, the scaling of signal variance depends on multiple factors but would be expected to increase with spot brightness, and to a lesser degree, size, both of which contribute to the overall integrated intensity

Fig S3. Scaling of fluorescence measurement noise with overall fluorescence intensity. Variance of mCherry fluorescence at a particular GFP fluorescence (red), from the dual-color interlaced reporter construct from Fig 3B, along with variance scaled by dividing out the mean mCherry fluorescence (black).

within the neighborhood.

The observed behavior of fluorescence variance is intriguing because previous work using the same spot detection methodology found that the dominant contributor to fluorescence noise was background fluorescence outside of the actively transcribing locus [25]. In contrast, this work is consistent with a scenario where the noise intrinsic to the individual fluorophore molecules dominates, leading to the observed scaling of fluorescent noise with the mean intensity. We speculate that, in this work, the difference in fluorescence noise behavior stems from the usage of mCherry, whose signal is lower, and therefore noisier, than that of GFP in the context of the fruit fly embryo (Fig 3F). In addition, other differences such as usage of MS2-mCherry instead of MS2-GFP and a different maternal fly line driving different levels of constitutive MCP-mCherry and PCP-GFP could change the relative strength of background fluorescence noise.

F Curation of inference results

Individual single cell inference results were filtered automatically and then run through an automated curation procedure for final quality control. First, due to experimental and computational imaging limits, some MS2 or PP7 trajectories were too short to run a meaningful inference on. As a result, we automatically skipped over any cell with an MS2 or PP7 signal with fewer than 30 datapoints. This amounted to 626 cells skipped out of a total of 1053, with 427 (41%) retained.

Second, the retained cells were run through an automated curation pipeline. For each single-cell fit, we calculated the average squared normalized residual δ^2, defined as

$$\delta^2 = \sum_{timepoints} \frac{(F_{data} - F_{fit})^2}{F_{data}^2},$$

where the summation occurs over all time points and F_{data} and F_{fit} correspond to the fluorescence data and fit, respectively. Thus, δ^2 gives a measure of how good or bad, on average, each single-cell fit is. S4A Fig and S4B Fig show histograms of the average squared normalized residual δ^2 for the entire $n = 427$ dataset, with log and linear x-axes. We see that the vast majority of data possesses values of δ^2 smaller than unity, with a long tail at higher values corresponding to bad fits. We decided to implement a cutoff of $\delta^2_{cutoff} = 1$ (red line), where any cell with a higher value of δ^2 was automatically discarded.

In sum, 355 cells of data were retained out of 427 total after this curation process. We reasoned that, since we still ended up with hundreds of single cells of data, the resultant statistical sample size was large enough to extract meaningful conclusions.

To assess the rejected fits for underlying biological causes, we did a qualitative examination for common features. There were several sources of bad fits. First, some traces possessed low signal-to-noise ratio (S4C Fig) that nevertheless yielded reasonable fits that were slightly above δ^2_{cutoff}. Still others simply had poor fits, possibly due to running into issues with the inference algorithm such as getting trapped in local minima (S4D Fig). We consider improvements to the algorithm to be outside the scope of this work, since the retained data still contain enough statistical size to provide interpretable results.

Finally, one potential biological source confounding the model could be the presence of substantial transcriptional bursting of the promoter. Although the majority of the traces we analyzed indicated that the *hunchback* reporter gene studied here possessed a promoter that was effectively ON during the cell cycle studied, a small fraction of traces (4% of the filtered cells) possessed substantial time dependence of the fluorescence signal, potentially resulting from rapid switching of the promoter between ON and OFF states (S4E Fig).

The presence of transcriptional bursts is of high biological significance, but capturing the behavior would require more specific models (e.g. two-state telegraph models like [35]). As a result, we relegate extensions of the model that can account for transcriptional bursting for future work. Thus, our work provides a self-contained framework applicable for describing the behavior of promoters that are primarily ON for the duration of the experiment and that do not experience transcriptional bursting.

Due to the variety of sources contributing to the rejected fits, we opted for a conservative approach and only analyzed the cells with high signal quality that did not exhibit the complications mentioned above. The number of retained fits were still much higher than the number of rejected fits (S4F Fig).

To check that the curation procedure did not incur substantial bias, we compared the average inferred mean initiation rate, elongation rate, and cleavage time as a function of embryo position between the post-filtering curated and uncurated datasets of size $n = 355$ and $n = 427$, respectively (S4G-I Fig). We observed no substantial difference between the two datasets, indicating that the curation procedure was not systematically altering the inference results.

Fig S4. Automated curation of data. (A, B) Histograms (blue) of average squared normalized residual of single-cell fits, in log (A) and linear (B) scale, with cutoff of $\delta^2_{cutoff} = 1$ shown in red in (B). (C) Example of bad fit from poor signal-to-noise ratio (SNR). (D) Example of bad fit of otherwise reasonable data from issues in fitting algorithm, for example due to local minima. (E) Example of bad fit due to potential presence of substantial bursting of promoter. (F) Number of single cell fits in each class of rejected fit, along with number of accepted fits, after the initial filtering based on number of time points. Altogether, 84% of filtered fits were accepted. The percentages of filtered fits the three rejected categories (low SNR, poor fits, bursting) were 7%, 5%, and 4%, respectively. The data shown in C-E are in each fluorophore's intrinsic arbitrary unit without rescaling, to present the fluorescence intensities in their raw form. (G, H, I) Comparison of average inferred (G) mean initiation rate, (H) elongation rate, and (I) cleavage time as a function of embryo position, between curated (blue) and uncurated (red) datasets. Values of δ^2 were 6.05, 1820, and 688 for the example fits shown in C-E, respectively, here given to illustrate the qualitative correspondence of δ^2 as a metric with the overall goodness-of-fit. Shading in G-I represent standard error of the mean for 355 and 427 cells across 7 embryos for curated and uncurated datasets, respectively.

G Validation of inference results

To assess the accuracy of the inference method, we validated our MCMC approach against a simulated dataset. Using the inferred distribution of model parameters from the experimental data, we generated a simulated dataset with our theoretical model (Section A in S1 File) and ran the MCMC inference on it.

The simulated dataset consisted of 300 cells. The model parameters used to simulate each individual cell's MS2 and PP7 fluorescences were drawn randomly from a Gaussian

distribution, with mean μ and standard deviation σ calculated from the distribution of inferred model parameters from the experimental data. Table A in S1 File shows the parameters used in the Gaussian distributions generating each single cell's model parameters. We chose to fix the time-dependent fluctuations in the initiation rate $\delta R(t)$ at zero since these fluctuations are not well understood at the single-cell level, and the *hunchback* reporter studied here is well parameterized by a mean initiation rate (Fig 2B).

	mean (μ)	standard deviation (σ)
$\langle R \rangle$	16.6 AU/min	5.1 AU/min
$\delta R(t)$	0	0
v_{elon}	1.8 kb/min	0.8 kb/min
τ_{cleave}	3.1 min	1.4 min
t_{on}	3.5 min	1.6 min
α	0.16	0.05
$MS2_{basal}$	10 AU	5 AU
$PP7_{basal}$	10 AU	5 AU

Table A. Mean and standard deviation of model parameters used in single-cell simulations.

In addition, fluorescence measurement error was generated for each single cell and at each time point by drawing a random number from a Gaussian distribution with mean 0 and standard deviation $10 \times \sqrt{F_{sim}}$ AU, where F_{sim} is the fluorescence at each time point, and adding this random number to the MS2 or PP7 fluorescence at that time point (prior to rescaling the MS2 fluorescence with the scaling factor α). Here, the $\sqrt{F_{sim}}$ factor in the magnitude of the fluorescence noise accounts for our observation that the variance of the fluorescence measurement noise scales linearly with the mean signal intensity (S3 Fig).

S5A Fig shows an example of the simulated MS2 and PP7 fluorescence from a single cell along with their corresponding fits. The resulting MCMC-sampled values of the mean initiation rate, elongation rate, and cleavage time are shown in the histograms in S5B Fig (blue), along with the ground truth for that single cell (red line). As described in Section D in S1 File, the mean value of each sampled distribution was retained for downstream statistical analysis.

The accuracy of the inference was investigated on three levels: 1) systematic errors affecting mean analyses, 2) random errors affecting measurements of distributions, and 3) spurious correlations between parameters affecting inter-parameter correlations.

First, the scaled error ε for each parameter was calculated on a single-cell basis as defined by

$$\varepsilon = \frac{x_{infer} - x_{truth}}{\mu_x},$$

where x represents the model parameter being investigated, the subscripts indicate whether the quantity is the inferred result or the ground truth for that single cell, and μ_x is the population mean of the parameter value from the experimental data (i.e., the values of the "mean" column in Table A in S1 File). For example, for the mean initiation rate $\langle R \rangle$, $\mu_{\langle R \rangle}$ takes the value 16.6 AU/min. ε gives a unitless measure of the magnitude of inference error of each single cell, where a value of 1 indicates an error that is as large as the population mean itself. Because the scaled error is defined as the error due to inference for a single cell, it is an intensive quantity that is independent from the overall dataset size.

S5C Fig shows the histogram of single-cell scaled errors $\varepsilon_{\langle R \rangle}$, $\varepsilon_{v_{elon}}$, and $\varepsilon_{tau_{cleave}}$ for the inferred mean initiation rate, elongation rate, and cleavage time, respectively. The

Fig S5. Overview of MCMC inference validation. (A) Example single-cell simulated data and inferred fits. (B) MCMC inference results for the simulated data in (A) for the mean initiation rate, elongation rate, and cleavage time. The histogram represents the raw MCMC sampled values, and the red line is the ground truth for this particular cell. The mean value of each histogram is then retained for further statistical analysis. (C) Scaled error of initiation, elongation, and cleavage for each simulated cell. (D) Comparison of relative magnitudes of random inference error and true experimental variability for the initiation, elongation, and cleavage parameters. (E, F, G, H) Single-cell correlations along with Spearman correlation coefficients and p-values for simulated data between (E) mean initiation rate and cleavage time, (F) mean initiation rate and elongation rate, (G) elongation rate and cleavage time, and (H) mean RNAP density and cleavage time, respectively. Blue points indicate single-cell values; black points and error bars indicate mean and SEM, respectively, binned across x-axis values. Line and shaded region indicate generalized linear model fit and 95% confidence interval, respectively. Linear fits were calculated using a generalized linear regression model and are presented for ease of visualization (see Methods and Materials for details).

majority of the scaled errors fall between -0.5 and 0.5, indicating that most inferred results possess relatively small error.

The systematic error on measurements of the ensemble mean can be estimated by calculating the mean of the scaled errors shown in S5C Fig. Doing so results in a value of -0.06 ± 0.01, -0.01 ± 0.02, and 0.04 ± 0.02 (mean and SEM) for the mean scaled error of the mean initiation rate, elongation rate, and cleavage time, respectively. For context, this means that, if the mean cleavage time is ~ 3 min, then the systematic error in the cleavage time is ~ 10 sec, about the time resolution of the data. Thus, the systematic error for each parameter is a couple orders of magnitude below that of the experimental mean value of each parameter, indicating that the inference provides an accurate and precise readout of the mean.

While the inference's systematic error across cells may be small, the presence of individual single-cell errors will affect measurements of distributions of parameters. To investigate the impact of these random errors, we quantified the fraction of total empirically inferred variability that consisted of inferential error. Specifically, for a parameter x, we separated the variance of single-cell measurements as

$$\sigma^2_{x,total} = \sigma^2_{x,empirical} + \sigma^2_{x,inference},$$

where $\sigma^2_{x,total}$ represents the overall single-cell variability observed in the data (the combination of empirical and inferential variability), $\sigma^2_{x,inference}$ represents the error inherent to our inference process, and $\sigma^2_{x,empirical}$ represents the true empirical variability after subtracting out inferential error $\sigma^2_{x,inference}$. Note that $\sigma^2_{x,total}$ is the square of the values in the standard deviation column in Table A in S1 File.

Dividing by the square of the population means μ_x yields

$$\frac{\sigma^2_{x,total}}{\mu_x^2} = \frac{\sigma^2_{x,empirical}}{\mu_x^2} + \frac{\sigma^2_{x,inference}}{\mu_x^2}.$$

Note that these are just squared CV terms, and that the last term is simply the square of the scaled error ε defined earlier

$$CV^2_{x,total} = CV^2_{x,empirical} + \varepsilon_x^2.$$

Thus, the overall impact of the inferential error can be quantified by calculating the relative magnitudes of the contributions of $CV^2_{x,empirical}$ and ε^2 to the total variability

$CV^2_{x,total}$. S5D Fig shows this separation, where the dark bars represent the squared scaled error ε^2, the light bars represent the true empirical variability $CV^2_{x,empirical}$, and the overall bars represent the total variability $CV^2_{x,total}$ obtained from the values of μ and σ in Table A in S1 File.

All three model parameters—initiation, elongation, and cleavage—possess no more than approximately 25% inferential error. Nevertheless, the presence of this much error indicates that measurements of distributions of these parameters will be somewhat confounded by the inherent error present in our inference method, highlighting the general difficulty in measuring values beyond the mean.

However, these errors in the inference of the variability of the transcription cycle parameters should not impact the results of investigating the distribution of elongation rates in Fig 4D, since the simulated results there were also pushed through the inference pipeline and should pick up similar inferential noise. Furthermore, the variances of the simulated distributions in the presence or absence of single-molecule elongation variability differed by essentially around a factor of two (S10D Fig), twice as much as the random error exhibited in the simulated results here (see Section M in S1 File for details).

Future improvements on increasing the accuracy of measurements of distributions could be achieved, for example, by utilizing interleaved loops such as those introduced in Fig 3B. Here, two orthogonal species of mRNA binding proteins fused to different fluorescent proteins would bind to interleaved loops located at the 5' end of the construct. In addition, a second pair of mRNA binding proteins would bind to an analogous set of interleaved loops located at the 3' end. The result would be a four-color experiment, with two colors reporting on transcription at the 5' end of the transcript, and two different colors reporting on transcription the 3' end. In this scenario, the data would provide independent readouts of the same underlying signal, making it possible to perform two independent inferences on the same nucleus. This would allow for the decomposition of the inference into biological variability and inferential error using techniques analogous to those presented in SectionK in S1 File.

Finally, we examined the inference method for spurious correlations to investigate the accuracy of the experimental single-cell correlations shown in Fig 5. The presence of spurious correlations would reflect inherent couplings in the inference method itself, since the simulation parameters were generated independently and stochastically.

S5E-H Fig show the single-cell correlations using the Spearman rank correlation coefficient between model parameters for the simulated dataset, as well as between the mean RNAP density and the cleavage time, as defined in the main text. Linear regression fits are also displayed for intuitive visualization. We discovered a slight positive correlation ($\rho = 0.15$) between the elongation rate and the cleavage time (S5G Fig, p-val $= 0.01$). In contrast, there was no significant correlation between the mean initiation rate and the cleavage time, the mean initiation rate and the elongation rate, and the mean RNAP density and the cleavage time (S5E, F, and H Fig). Although the relationship between the elongation rate and the cleavage time possessed the same, albeit weaker, correlation as found in the data (Fig 5C), the main finding in the main text of the correlation between the mean RNAP density and the cleavage time was not reproduced by the simulations (S5H Fig). The comparisons of Spearman rank correlation coefficients and p-values between the data and simulations are summarized in Table B in S1 File.

Thus, our results validated the single-cell correlations discovered in the main text, indicating that the experimental results were not the product of spurious correlations.

	initiation/cleavage	initiation/elongation	elongation/cleavage	RNAP density/cleavage
data	$\rho = -0.52$<br>p-val ≈ 0<br>negative correlation	$\rho = -0.21$<br>p-val $= 5 \times 10^{-5}$<br>negative correlation	$\rho = 0.35$<br>p-val $= 2 \times 10^{-11}$<br>positive correlation	$\rho = -0.55$<br>p-val ≈ 0<br>negative correlation
simulation	$\rho = 0.07$<br>p-val $= 0.24$<br>insignificant correlation	$\rho = 0.01$<br>p-val $= 0.86$<br>insignificant correlation	$\rho = 0.15$<br>p-val $= 0.01$<br>positive correlation	$\rho = -0.01$<br>p-val $= 0.86$<br>insignificant correlation

Table B. Comparison of Spearman rank correlation coefficients and p-values between experimental and simulated single-cell correlations.

H Validation of the RNAP processivity assumption

The calibration between the MS2 and PP7 signals (Fig 3) provided an opportunity to test the processivity assumption presented in the main text, namely that the majority of loaded RNAP molecules transcribe to the end of the gene without falling off. To estimate the processivity quantitatively, we assume that a series of N RNAP molecules transcribes past the MS2 stem loop sequence at the 5' end of the reporter gene, and that only pN successfully transcribe past the PP7 stem loop sequence at the 3' end. Here, we define p to be the processivity factor, and require $0 < p < 1$. Thus, $p = 1$ indicates maximal processivity where every RNAP molecule that transcribes the MS2 sequence also transcribes the PP7 sequence, and $p = 0$ indicates minimal processivity, where no RNAP molecules make it to the PP7 sequence.

We assume that no RNAP molecules fall off the gene while they transcribe the interlaced MS2/PP7 loops used in the calibration experiment described in Fig 3B. Under this assumption, N RNAP molecules will fully transcribe both sets of stem loop sequences, allowing us to define the scaling factor as the ratio of total fluorescence values

$$\alpha_{\text{calib}} = \frac{NF_{MS2}}{NF_{PP7}} = \frac{F_{MS2}}{F_{PP7}}.$$

Note that, in this simple model, RNAP molecules can still fall off the gene after they transcribe the set of MS2/PP7 loops. Now, we consider the construct with MS2 and PP7 at opposite ends of the gene used in the main text. Allowing a fraction p of RNAP molecules to fall off the gene between the MS2 and PP7 loops, we arrive at a scaling factor

$$\alpha_{\text{infer}} = \frac{NF_{MS2}}{pNF_{PP7}} = \frac{F_{MS2}}{pF_{PP7}}.$$

We can thus calculate the processivity p from taking the ratio of the true and biased scaling factors

$$p = \frac{\alpha_{\text{calib}}}{\alpha_{\text{infer}}}.$$

Taking the mean value of α_{calib} from our control experiment using the interlaced MS2/PP7 loops to be the true value and the mean value of α_{infer} from the inference from the main text to be the biased value, we calculate a mean processivity of $p = 0.96$, with a negligible standard error of 4.81×10^{-5}. Thus, on average, 96% of RNAP molecules that successfully transcribe the 5' MS2 stem loop sequence also successfully transcribe the 3' PP7 stem loop sequence, confirming previous results [25, 76] and lending support to the processivity assumption invoked in our model.

Fig S6. Comparison of intra- and inter-embryo variability for inferred (A) mean initiation rates, (B) elongation rates, and (C) cleavage times, as a function of embryo position. (D) Intra- and inter-embryo variability for transcriptional parameters averaged across all embryo positions. (A-C, lines and shaded regions indicate mean and standard error of the mean, respectively; D, error bars indicate bootstrapped standard error error across 100 bootstrap samples. Data were taken over 355 cells across 7 embryos, with approximately 10-90 cells per embryo in the region of the embryo examined here.)

Fig S7. Single cell distributions of inferred parameters. (A-C) Full single-cell distributions of (A) mean initiation rate, (B) elongation rate, and (C) cleavage time as a function of embryo position.

I Comparing intra- and inter-embryo variability

In the analysis in the main text, we treated all single cell inference results equally within one statistical set. In principle, this is justified only if the variability between single cells is at least as large as the variability between individual embryos. In this section we prove this assumption.

Here, we examine two quantities: the *intra-embryo variability*, defined as the variance in a parameter across all single cells in a single embryo, and the *inter-embryo variability*, defined as the variance across embryos in the single-embryo mean of a parameter. We examined these two quantities for the three primary inferred parameters—the mean initiation rate, elongation rate, and cleavage time.

S6A-C Fig shows the results of this comparison as a function of embryo position, where the red (blue) lines indicate the intra- (inter-) embryo variability and the red (blue) shaded regions indicate the standard error (bootstrapped standard error) in the intra- (inter-) embryo variability. For all of the parameters, the intra-embryo variability is at least as large as the inter-embryo variability, validating our treatment of all of the single-cell inference results as a single dataset, regardless of embryo.

This is seen more clearly when the data are averaged across embryo position. As shown in S6D Fig, the inter-embryo variability of each parameter is substantially higher than the intra-embryo variability.

J Full distributions of transcriptional parameters as a function of embryo position

Fig 4 presents inferred values of the transcriptional parameters in the form of population means and CVs as a function of embryo position. We chose this form of presentation to focus on spatial variation of these parameters via a succinct visualization.

S7 Fig shows the full distributions of the transcriptional parameters as a function of embryo position. For each parameter, the observed variability at a particular position in the embryo is quite broad, indicating substantial cell-to-cell variability. Nevertheless, there is no clear indication of multimodal behavior, indicating that the mean is still a reliable metric of population-averaged behavior.

K Comparison of variability in mean initiation rate reported by our inference with static measurements

A widespread strategy to measure variability in transcription initiation relies on techniques such as single-molecule FISH (smFISH), which count the number of nascent transcripts at a transcribing locus in a fixed sample [39–41, 43, 45, 48–52, 56–58, 76–78, 80–83]. These single time point measurements are typically interpreted as reporting on the cell-to-cell variability in transcription initiation. Further, under the right conditions, the variability reported by this method has been shown to be dominated by biological sources of variability and to have a negligible contribution from experimental sources of noise [57].

Inspired by these measurements in fixed embryos, we sought to determine how well our approach could report on biological variability. To do so, we contrasted the inference results of the transcriptional activity of our *hunchback* reporter with a snapshot-based analysis inspired by single-molecule FISH [57]. Specifically, we calculated the CVs in the raw MS2 and PP7 fluorescence in snapshots taken at 10 minutes after the start of nuclear cycle 14, from the same post-curation cells analyzed with the inference method. We reasoned that, since this calculation does not utilize the full time-resolved nature of the data, it provides a baseline measurement of total noise that encompasses both experimental and biological variability. As a point of comparison, we also calculated the CV in the instantaneous MS2 signal from another work using a similar P2P-MS2-lacZ construct [61].

S8A Fig shows the CV as a function of embryo position as reported by these different approaches. For the static measurements (red, green, and blue), the CV values lay around 20% to 80%. The CV of the inferred mean initiation rate (purple) exhibited similar values, although it was slightly lower in a systematic fashion. This difference was likely due to the fact that the inference relies on time-dependent measurements that can average out certain sources of error such as experimental noise, whereas such time averaging is not possible in the context of single time point measurements.

To succinctly quantify variability in the mean initiation rate, we then calculated the position-averaged squared CV for the same measurements in S8A Fig. The resulting squared CV values are shown in S8B Fig. Although the static measurements possessed essentially identical squared CVs (blue, red, green), the inference method exhibited a clear reduction in the squared CV (purple).

To test whether the discrepancy in the variability between time-resolved and snapshot-based measurements arose from differences in the experimental error of each technique, we used the formalism introduced by [84] to separate the noise in the system into uncorrelated and correlated components. Here, uncorrelated noise represents random measurement error, while correlated noise contains both systematic measurement error as well as true biological variability. To perform this separation, we utilized the alternating MS2-PP7 reporter used in the calibration calculation (Fig 3B). Because the MS2 and PP7 fluorescent signals in this reporter construct should, in principle, reflect the same underlying biological signal, deviations in each signal from each other should report on the relative magnitudes of both types of noise.

First, we defined the deviations δ_{MS2} and δ_{PP7} of each instantaneous MS2 and PP7 fluorescent signal from the mean MS2 and PP7 fluorescence signals, averaged across

nuclei and time

$$\delta_{MS2} = \frac{F_{MS2}}{\langle F_{MS2} \rangle} - 1$$
$$\delta_{PP7} = \frac{F_{PP7}}{\langle F_{PP7} \rangle} - 1,$$

where F_{MS2} and F_{PP7} are the respective instantaneous MS2 and PP7 fluorescence values for a given nucleus and time point, and $\langle F_{MS2} \rangle$ and $\langle F_{PP7} \rangle$ are the respective mean MS2 and PP7 fluorescence values, averaged across nuclei and time points. Using these deviations, the uncorrelated and correlated noise terms are defined as

$$\eta^2_{uncorr} = \frac{1}{2} \langle (\delta_{MS2} - \delta_{PP7})^2 \rangle$$
$$\eta^2_{corr} = \langle \delta_{MS2} \delta_{PP7} \rangle,$$

where the brackets indicate an ensemble average over time points and cells [84]. From this, the total noise η^2_{tot}, defined as the variance σ^2 divided by the mean squared μ^2, is simply the uncorrelated and correlated noise components added in quadrature

$$\eta^2_{tot} = \frac{\sigma^2}{\mu^2} = \eta^2_{uncorr} + \eta^2_{corr}.$$

Note that the total noise η^2_{tot} is simply the squared coefficient of variation. Thus, the squared coefficient of variation (CV^2) of our data is equal to η^2_{tot} and can be separated into the uncorrelated and correlated components.

S8B Fig shows this CV^2 (averaged across all embryo positions) for snapshots of the interlaced loop construct compared with the separated uncorrelated and correlated noise sources. Intriguingly, the uncorrelated and correlated noise (yellow) each contribute about half to the overall noise. We posit that the relative magnitude of partitioning between correlated and uncorrelated noise also holds for the static measurements of spot fluorescence (S8B Fig, blue, red and green). As a result, given this assumption, we can calculate the correlated and uncorrelated variability contributions to total squared CV from these static measurements. This is shown in light and dark red in the case of the static MS2 fluorescence measurement in S8B Fig. The plot reveals that the correlated noise component of the static measurements (dark red) is only slightly smaller than the overall noise measured by the inference (purple) , suggesting that our inference method primarily reports on correlated variability.

As a result, the MCMC inference method can quantitatively capture the true biological variability in the mean initiation rate while separating out most of the uncorrelated contribution due to random experimental noise. Thus our results support the power of model-driven inference approaches in providing clean readouts of variability in transcriptional parameters.

L Comparison of distribution of elongation rates with other works

As an additional validation of our inference results, we compared the distribution of single-cell inferred elongation rates with those reported in two similar works by [23] and [26]. Both of these works used a two-color live imaging reporter like the one utilized in this work, and measured the time delay between the onset of each stem loop signal to estimate a single-cell mean elongation rate. [26] studied a similar *hunchback* reporter to the one used here, while [23] used a reporter construct in yeast.

Fig S8. Comparison of coefficients of variation (CV) between inferred mean initiation rates and instantaneous counts of number of nascent RNA transcripts. (A) Position-dependent CV of inferred mean initiation rate (purple) compared with static measurements of MS2 and PP7 raw fluorescence (red, green) from the dual-color reporter (Fig 1C), as well as with static measurements of MS2 data from [61] (blue). (B) Position-averaged squared CVs of the same measurements, where the entire dataset is treated as a single sample and embryo position information is disregarded. In addition, separation of uncorrelated and correlated sources of variability are shown, calculated using the reporter described in Fig 3B. (A, Shaded regions indicate bootstrapped standard error of the mean; B, error bars indicate bootstrapped standard error of the mean for $n = 100$ bootstrap samples.)

Fig S9. Comparison of distribution of elongation rates (green) with previous studies ([23], red and [26], blue). Distributions of previous studies were adapted from Figs. 2D and 2A of [23] and [26], respectively.

S9 Fig shows the comparison of distributions of elongation rates. Because the reporter constructs and analysis techniques differed between works, a quantitative comparison is not possible. Nevertheless, all three sets of results report a significant cell-to-cell variability in mean elongation rate, ranging from 1 kb/min to 3 kb/min.

M Theoretical investigation of single-cell distribution of elongation rates

To investigate the molecular mechanisms underling single-cell distributions of elongation rates obtained from the inference, we developed a single-molecule theoretical model. We were interested in how the observed variability in single-cell elongation rates could constrain models of the single-molecule variability in RNAP elongation rates. To disregard effects due to position-dependent modulations in the transcription initiation rate, we only studied cells anterior of 40% along the embryo length, where the initiation rate was roughly constant.

The model was adapted from the stochastic Monte Carlo simulation used in [92], which accounts for the finite size of RNAP molecules (S10A Fig). Here, single RNAP molecules are represented by one-dimensional objects of size $N_{footprint}$ that traverse a gene consisting of a one-dimensional lattice with a total number of sites, corresponding to single base pairs, equal to N_{sites}. The position of the active site of molecule i is given by x_i, which takes integer values—each integer corresponds to a single base pair of the gene lattice. Because RNAP molecules have a finite size, given by $N_{footprint}$, an RNAP molecule i thus occupies the lattice sites from x_i to $x_i + N_{footprint}$. In this model, we do not incorporate sequence-dependent RNAP pausing along the gene.

New RNAP molecules are loaded at the start of the gene located at $x = 0$. Due to the exclusionary interactions between molecules, simultaneously simulating the motion of all molecules is unfeasible, and a simulation rule dictating the order of events is necessary.

At each simulation timestep dt, a randomized sequence of indices is created from the following sequence

$$\mathcal{I} = \{0, 1, \ldots, N\},$$

where $\{1, \ldots, N\}$ correspond to any RNAP molecules $i = 1, \ldots, N$ already existing on the gene, and 0 corresponds to the promoter loading site that generates new RNAP molecules.

Choosing indices i from the random sequence $\mathcal{I}$ obtained above, the following actions are taken. If the index i indicates that an RNAP molecule was chosen ($i > 0$), then that RNAP molecule advances forward with stochastic rate ϵ. This probability is simulated by drawing a random number from a Poisson distribution with parameter $\epsilon\ dt$, thus giving an expected distance traveled of $\epsilon\ dt$ per timestep (recall that, for a Poisson distribution with parameter $\epsilon\ dt$, the resulting random variable corresponds to the number of occurrences in a time frame dt.). If this movement would cause the RNAP molecule to overlap with another RNAP molecule, then no action is taken. Otherwise, the RNAP molecule moves forward the number of steps given by the generated random variable.

If no RNAP molecule on the gene is chosen ($i = 0$), an RNAP molecule is loaded using a probability parameterized by the term $\beta\ dt$, only if no already existing RNAP molecules overlap with the footprint of the new RNAP molecule. If such an overlap occurs, then no action is taken. Otherwise, to calculate the probability of loading, a random number is drawn from a Poisson distribution with parameter $\beta\ dt$. If this number is one or higher, then the loading event is considered a success. The process is repeated until a total simulation time T has elapsed.

To simulate potential single-molecule variability, each RNAP molecule can possess a different stepping rate ϵ. For a given RNAP molecule i, its stochastic stepping rate ϵ_i is drawn from a truncated normal distribution Tr with mean μ_ϵ and standard deviation σ_ϵ and lower and upper limits 1 and infinity bp/sec, respectively

$$\epsilon_i = Tr(\epsilon, \sigma_\epsilon, 0, \infty).$$

Once the position of the active site of an RNAP molecule exceeds that of the total number of sites N_{sites}, i.e. the molecule reaches the end of the gene, it is removed from the simulation after the cleavage time τ elapses..

Finally, to account for single-cell variability in the transcription initiation rate, the loading rate β and cleavage time τ were allowed to vary across each simulated cell j by drawing these magnitudes from a Gaussian distribution with parameters reflecting the actual data. Since *hunchback* is known to load new nascent RNA transcripts at a rate of 1 molecule every 6 seconds in the anterior of the embryo [25], we thus chose the mean of this distribution μ_β to be 1 molecule/6 $s = 0.17\ s^{-1}$. The standard deviation σ_β was chosen to be this mean multiplied by the CV in the initiation rate in the anterior inferred in the main text, resulting in a value of 0.05 s^{-1}. Thus, for simulated cell j

$$\beta_j = N(\mu_\beta, \sigma_\beta),$$

where any negative value was replaced with zero.

Similarly, the cleavage time τ_j for each simulated cell was drawn from a Gaussian distribution with mean $\mu_\tau = 2.5$ min and standard deviation $\sigma_\tau = 1.6$ min. These values were obtained from the distribution of inferred cleavage times in the anterior of the embryo. The values of each simulation parameter are summarized in Table C in S1 File.

From these simulations, the positions of each RNAP molecule on the gene as a function of time were saved and then fed into the model of the reporter gene (Section A in S1 File), producing simulated single-cell MS2 and PP7 fluorescence traces (S10B Fig). Simulated fluorescence noise was added using the same parameters as in the validation simulations discussed earlier (Section G in S1 File, Table A in S1 File, and S5 Fig). These fluorescence traces were then run through the inference pipeline (Section D in S1 File), resulting in inferred distributions of single-cell mean elongation rates from the single-molecule elongation simulation.

In order to compare these results with the empirically inferred distribution of elongation rates (Fig 4D, red), we first considered a scenario where the single-molecule variability in stepping rates σ_ϵ was fixed at zero and the mean stepping rate μ_ϵ was

Parameter	Description	Value
T	total simulation time	600 sec
dt	simulation timestep	0.5 sec
N_{sites}	size of lattice	6626 bp
$N_{footprint}$	RNAP footprint [105]	40 bp
μ_β	mean loading rate	0.17 sec^{-1}
σ_β	standard deviation of loading rate	0.05 sec^{-1}
μ_τ	mean cleavage time	2.5 min
σ_τ	standard deviation of cleavage time	1.6 min
μ_ϵ	mean elongation rate	free parameter
σ_ϵ	standard deviation of elongation rate	free parameter

Table C. Parameters used in single-molecule Monte Carlo simulation of elongation rates.

varied from 0.6 to 2.1 kb/min. While the combination of exclusionary interactions between RNAP molecules, stochasticity in single-molecule stepping, and inferential noise did produce some cell-to-cell variability (S10C Fig, top row), the resulting distributions nevertheless were unable to reproduce the large variance observed in the data. This can be seen by plotting the mean and variance of the simulated distributions (S10D Fig, blue), where we see that the variance in the case of $\sigma_\epsilon = 0$ is always below that of the data (S10D Fig, purple).

Next, we allowed σ_ϵ to vary, simulating small to moderate variability with values of $\sigma_\epsilon = 0.3$ kb/min and $\sigma_\epsilon = 0.6$ kb/min. As expected, this single-molecule variability caused the inferred single-cell elongation rate distributions to widen (S10C Fig, middle and bottom rows). In the presence of this variability, there existed parameter sets where the mean and variance of the simulated distributions quantitatively matched the empirical distribution within error (S10D Fig, red and gold).

The distributions presented in the main text correspond to the following parameter values. For the case with no molecular variability in elongation rates (Fig 4D, brown), we used $\mu_\epsilon = 0.9$ kb/min and $\sigma_\epsilon = 0$ kb/min, chosen as the simulated parameter set with results closest to the inferred mean and variance of empirical elongation rates (S10D Fig, lower black arrow). For the case with molecular variability in elongation rates (Fig 4D, gold), we used $\mu_\epsilon = 0.9$ kb/min and $\sigma_\epsilon = 0.3$ kb/min, chosen as a representative example of a simulation possessing a mean and variance in elongation rate that agreed with the inferred mean and variance of empirical elongation rates within error (S10D Fig, upper black arrow), as well as qualitatively agreeing with the inferred distribution (Fig 4D, gold).

N Single-cell correlation analysis using full posterior distributions

The single-cell inter-parameter correlations presented in the main text (Fig 5 were based off of mean values from the posterior distributions obtained from the inference procedure for ease of interpretation and visualization. In principle, these correlations could possess high amounts of uncertainty due to uncertainty in the single-cell parameter estimates. Here, we conduct a correlation analysis based on the full posterior distributions from the inference and validate the mean results presented in the main text.

To do so, we used a Monte Carlo simulation to construct a distribution of Spearman correlation coefficients and investigated if the mean Spearman correlation coefficients presented in Fig 5 agreed with these simulated distributions.

Fig S10. Single-molecule simulations of elongation dynamics require molecular variability to describe empirical distributions. (A) Cartoon overview of simulation. RNAP molecules with footprint $N_{footprint}$ stochastically advance along a one-dimensional gene represented as a lattice with N_{sites} unique sites, with each site equivalent to a single base pair. Each RNAP molecule i possesses an intrinsic stepping rate ϵ_i, and each cell j stochastically loads new RNAP molecules at the promoter with rate β_j and cleaves finished RNAP molecules after a cleavage time τ_j. (B) Sample simulated MS2 and PP7 fluorescence traces for a single cell, using the single-molecule simulation with parameters $\mu_\epsilon = 1.8$ kb/min and $\sigma_\epsilon = 0$ kb/min, along with inferred fits. (C) Simulated distributions of elongation rates (red) for varying values of μ_ϵ and σ_ϵ, compared with inferred empirical distribution from data (blue). (D) Mean and variance of simulated and empirical distributions of elongation rates for varying values of μ_ϵ and σ_ϵ. Without enough variability in the elongation rate of individual RNAP molecules (blue), the single-molecule model cannot produce the variance observed in the data (purple). However, in the presence of enough molecular variability, the empirical distribution's mean and variance can be reproduced for certain parameter sets (red and gold). Black arrows correspond to parameter sets used for simulated distributions presented in the main text (Fig 4D).

First, we extracted the mean and variance of the inferred posterior distribution obtained from each single cell, for each transcriptional parameter (Fig 2C and E). We then simulated $N = 50,000$ new single-cell datasets comprising the mean initiation rate, elongation rate, and cleavage time, where these values were generated from Gaussian distributions parameterized by the means and variances from each parameter's posterior distribution at the single-cell level.

Thus, each of the $N = 50,000$ simulations resulted in a simulated dataset of $n = 355$ cells with randomly generated transcriptional parameter values obtained from the information inside the single-cell inferred posterior distributions from the experimental data. We then calculated an individual Spearman correlation coefficient and associated p-value for each simulation, generating an $N = 50,000$ distribution for each correlation relationship.

S11A Fig and S11B Fig show the ensuing distribution of p-values for the Spearman correlation coefficient between the mean initiation rate and elongation rate, as well as between the elongation rate and cleavage time, respectively. The p-values for the relationships between the mean initiation rate and cleavage time and between the mean RNAP density and cleavage time were essentially zero due to floating point error. Thus, the distributions of p-values for all four inter-parameter relationships were extremely small and support the statistical significance of their associated correlations.

S11C Fig shows the simulated distributions of Spearman correlation coefficients for all four relationships (histograms), along with the values obtained from the simpler mean analysis presented in the main text (dashed lines). We see that using the full posterior via this Monte Carlo simulation yields distributions that are in agreement with the results from the mean analysis, and that the distributions themselves are narrow, with widths of around 0.05. As a result, the correlations obtained from utilizing only mean inferred parameters quantitatively agree with the results obtained from utilizing the full Bayesian posterior obtained from the MCMC inference procedure.

Thus, our original analysis is robust, and we chose to retain its presentation in the main text for simplicity and ease of understanding.

Fig S11. Monte Carlo simulation of error in single-cell analysis. (A, B) p-values of Spearman correlation coefficient for relationships between mean initiation rate and elongation rate (A) and between elongation rate and cleavage time (B). The p-values for the relationships between mean initiation rate and cleavage time as well as between mean RNAP density and cleavage time were essentially zero due to floating point error. (C). Distributions of Spearman correlation coefficients between mean initiation rate and cleavage time (blue), mean initiation rate and elongation rate (red), elongation rate and cleavage time (green), and mean RNAP density and cleavage time (purple). Results from mean-level analysis (Fig 5) are shown in dashed lines.

O Supplementary Videos

S1. **Video 1. Measurement of main reporter construct.** Movie of P2P-MS2-lacZ-PP7 reporter construct used in an embryo in nuclear cycle 14. Fluorescence intensities are maximum projections in the z-plane. Time is defined with respect to the previous anaphase.

S2. **Video 2. Measurement of interlaced reporter construct.** Movie of P2P-24x(MS2/PP7) reporter construct used in an embryo in nuclear cycle 14. Fluorescence intensities are maximum projections in the z-plane. Time is defined with respect to the previous anaphase.
