# Supplementary material for: Real-time single-cell characterization of the eukaryotic transcription cycle reveals correlations between RNA initiation, elongation, and cleavage
Source: PLoS Comput Biol. 2021 May 18;17(5):e1008999. doi: 10.1371/journal.pcbi.1008999 (PMC8162642; doi:10.1371/journal.pcbi.1008999)
Supplement: Attachment — Submitted filename: Review.pdf [file pcbi.1008999.s016.pdf]

Review for "*Single-cell characterization of the eukaryotic transcription cycle using live imaging and statistical inference*" by Liu *et al.*

Summary:

In this work, Liu *et al.* present a novel computational technique to simultaneously infer the effective parameters of the transcription cycle (including initiation, elongation, and cleavage of the nascent transcript) at the single-cell level from live-imaging of transcription using a two-color MS2/PP7 reporter gene. The authors apply this technique to study these parameters in developing fruit flies by analyzing the dynamics of ms2/pp7 gene controlled by the hunchback P2 promoter. From the fitted parameters' distributions, the authors show significant variability in the elongation rates between transcribing RNAP and a small negative correlation between the transcription initiation rate and RNA cleavage time. The results are extensively compared with findings from previous works on transcription initiation, elongation and termination.

MS2 and PP7 reporter genes have been shown to be powerful tools to study the *in vivo* dynamics of transcription in many organisms, from bacteria to human. The combination of the two reporter systems is promising in revealing the parameters of transcription cycle, as demonstrated in the studies of e.g. elongation rates (Fukaya et al., 2017), RNA splicing kinetics (Coulon et al., 2014). Due to the fact that the fluorescence signals are usually from multiple nascent RNAs, a computational framework to extract the kinetic parameters of transcription cycle, as intended in this work, is welcomed.

However, in this work, the model's assumptions are not adequately justified. The inference method, despite its flexibility to account for more complex models, is undermined by the arbitrary hierarchical fitting method. When applying the inference to individual traces, the systematic error is shown to be significant enough to affect the conclusions, especially regarding the parameters' variability and correlation.

As these issues are at the bottleneck of this work, it is difficult to evaluate the validity of the subsequent presentations and interpretations of the inference results from MS2/PP7 data, despite being well written in details.

Major points:

1) As the conclusions are drawn entirely by fitting a 3-step model (line 92-107) to a single dataset of hunchback P2 reporter, the presented model needs to be justified first. Without this, it is difficult to conclude about the steps at the mechanistic level.

-How the cleavage time can be distinguished from RNAP pausing at random or specific sites of the reporter gene (Herbert et al, Cell 2006)? Please clarify on the possible time scales of these steps to justify the preference of the cleavage time. I would also like to see how this technique can help in model selections.

-Transcription in eukaryotes, particularly in developing flies, has been shown to be very bursty. In *Drosophila* embryogenesis, the inferred ON-OFF periods are found to be ~ 1-5 minutes (Lammers et al. PNAS 2019, Desponds et al. PLOS CB 2016, Bothma et al. 2014), of the same order as the elongation time of MS2/PP7 transcript in this study (τ_{dwell}). Is this bursty dynamics accounted for in

the temporal RNAP firing rate $R(t)$? If not, how does including bursty behavior affect the final conclusions?

2) Please clarify on the model:

-The fluctuation term $\delta R(t)$: is it free vector (arbitrary) term, correlated noise or uncorrelated noise? From the SI, it appears to be uncorrelated noise as its parameters are not inferred. Please clarify in the main text.

-If $\delta R(t)$ is uncorrelated noise, the fluorescent signal should be deterministic (as in Fig. 1). Why there are fluctuations in the prediction of the model (Fig. S3, Fig. S4) or the downward trend in Fig. 2B after 10 minutes.

-Does $R(t)$ account for the promoter bursty dynamics (Desponds et al, PLOS CB, 2016) or only dynamics of initiation during the ON period (a single burst)? How would this bursty dynamics affect the fluorescent traces?

-Do you assume Gaussian noise on top of the signal from gene loci?

3) The hierarchical fitting method seems confusing and arbitrary. First, the authors show that the inference from some longer (18 minutes) traces does not fit well with its early time points (line 870). For these traces that are not well fitted, only shorter (10 minutes) traces are fed to the inference (line 875). Then, the authors show that the refitted model from the short traces can generate traces that capture the dynamics in longer traces (line 885).

-In principle, it is the model's fault if it could not explain the data. For example, the promoter can turn OFF, leading to changes in loci fluorescent intensity after the initial uphill slope. I think data treatment for all traces should be done BEFORE the fit, rather than after the fit and only to a subset of traces.

-The model that fits the short traces is shown to capture the longer traces' dynamics, which were not well fitted before. Intuitively, this suggests a bias in the inference from individual traces, as demonstrated with simulated data.

-When testing the inference on simulated data (Fig. S4C), the scaled error (or bias per cell) distribution is symmetric indicating that the estimation of the ensemble mean parameter values may be correct. However, the scaled error's ranges of -0.5 to 0.5 should be considered significant, as it is of the same order as the CV of the inferred parameter distribution (Fig. 2). Therefore, conclusions on the variability of the elongation rates should all be reconsidered.

-Does the scaled error reduce with more nuclei/embryo?

4) Regarding the MCMC inference method, for each cell, after extracting the posterior distribution of the parameters, why the mean value is retained, rather than the mode (best fit)?

5) I would like to see more discussion on the findings (e.g. variability in the elongation rates, RNAP crowding at termination site) and which additional experiments (e.g. additional ms2/pp7 configurations) that can, in complement with this approach, validate these findings.

Minor:

-Until reading the detailed model section of the Appendix, I had the impression that elongation times of ms2 and pp7 stemloops (~1kb?) are considered negligible. Please add the length in base pair of the ms2, pp7 stem loops, lacZ and lacY in Fig. 1C, or at least some text in the main text to clarify.

-Figure 1D, panel iii, left: R should represent the height of the slope, not the duration.

-Figure 2B: it is not clear whether the data points are taken from discrete uniform time interval. When active loci is not detected, do you assign a zero intensity value or the background value?

-Readers would benefit from Fig. S1C being moved to the main text as it provides intuitions on how each parameter can affect the final trace.

-100 cells for the evaluation of the inference seems very small, given the number and variability in distribution of the input parameters.
