# Supplementary material for: Real-time single-cell characterization of the eukaryotic transcription cycle reveals correlations between RNA initiation, elongation, and cleavage
Source: PLoS Comput Biol. 2021 May 18;17(5):e1008999. doi: 10.1371/journal.pcbi.1008999 (PMC8162642; doi:10.1371/journal.pcbi.1008999)
Supplement: Attachment — Submitted filename: TranscriptionCycle_PLoS_Response.pdf [file pcbi.1008999.s017.pdf]

Reviewer's Responses to Questions

Comments to the Authors:

Please note here if the review is uploaded as an attachment.

We thank the reviewers and reviewing editor for their comments. We've copied these comments below and have responded to them in-line, point-by-point. Where appropriate, we've incorporated these critiques and suggestions as new content into the manuscript, which we believe is much improved with the addition of this feedback.

Reviewer #1:

Regulation of transcription is a fundamental problem that has received growing attention with the emergence of technologies that enable single-cell measurements. In their manuscript, Liu et al hybridize experimental and computational approaches to investigate single cell variability of biophysical parameters for the transcription cycle across the AP axis of developing fruit fly embryos. Nascent mRNA production is quantified over time in living cells using orthogonal fluorescence-labeled stem loops placed at the 3' and 5' ends of a transgenic reporter gene. Biophysical parameters are subsequently calculated for each cell using Bayesian inference and mechanistic modeling. The resultant fits reveal significant cell-to-cell variability in rates for transcriptional initiation, elongation, and cleavage, and also support a range for intermolecular variability between individual RNA polymerases. Value in the approach for hypothesis generation/refinement is furthermore demonstrated with parameter co-variation analysis, suggesting novel mechanistic relationships between steps of the transcription cycle that are likely to be the topic of follow-up studies. Although the manuscript can still be improved by addressing several issues, it is elegant in its simplicity and of high technical quality. Overall, I expect the optimized approach and results will valuable to the readership of PLoS Computational Biology and should be published following revision.

Author response: We thank the reviewer for their assessment. We have responded to their comments below, and believe that the manuscript is greatly improved after incorporating the suggestions and critiques presented.

Major Comments:

1. My predominant concern relates to interpretation of the data. In supplementary movie 1, it’s clear that mCherry peaks in intensity and fades before the eGFP channel approaches its peak in intensity in the same cell (further supported by the data points for a single cell in figure 2b). This seems in contrast with the assumptions of RNA processivity and instantaneous cleavage in timescales of the experiment. Based on these assumptions my expectation is that decay in intensity for both fluorescent molecules should begin and proceed simultaneously. Is there an interpretation of this phenomenon that does not conflict with the assumptions?

Author response: The reconciliation between the reviewer’s assumptions with the observed decay in intensity in fluorescence lies in the fact that there is a slight time dependence in the transcriptional dynamics of the *hunchback* gene. Specifically, the initiation rate slowly decays from a maximal ON state to an OFF state starting around ~10-18 min into the nuclear cycle (previously observed by Garcia et al. 2013, *Current Biology* and Liu et al. 2013, *PLoS ONE*). Thus, if the promoter turns off at some point, there will still be some finite time for freshly initiated RNAP molecules to traverse the gene and finish transcribing. Because the MS2/mCherry readout is 5’ of the PP7/GFP readout, it will decay in intensity first when no new RNAP molecules transcribe its stem loops while recently initiated RNAP molecules still have yet to reach the PP7 stem loops. This is illustrated in the cartoon in Fig. 1D, where after the initiation rate drops to zero, the MS2 signal begins decaying before the PP7 signal. Note that here, because the cleavage process occurs before the promoter shuts off, the two signals plateau together but exhibit a temporal delay in the decay.

For the specific case pointed out by the reviewer where the MS2 signal peaks before the PP7 signal, consider the cartoon shown in Fig. R1. In contrast to Fig. 1D, here, the cleavage process occurs after the promoter shuts off, resulting in the MS2 signal plateau first, and then the PP7 signal due to the temporal delay between the 5’ and 3’ stem loop sequences.

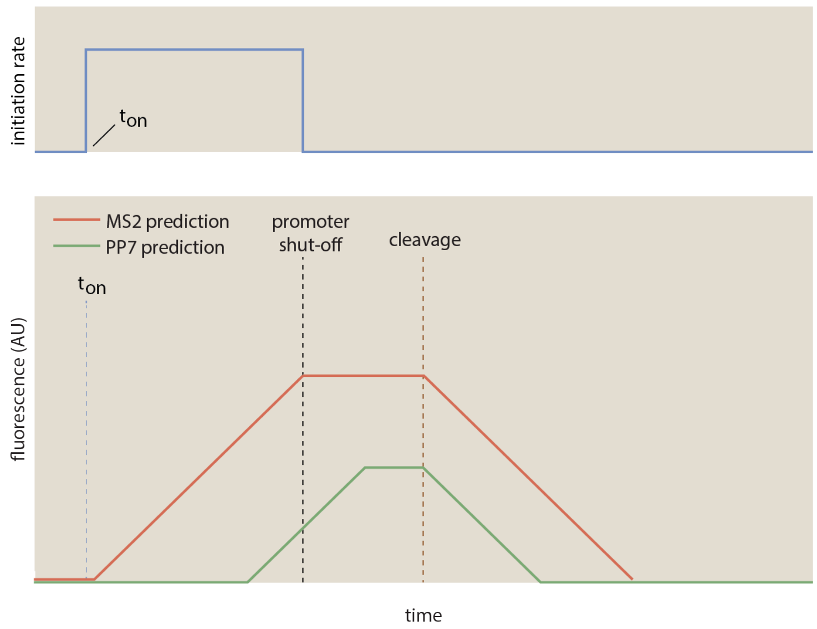

Figure R1 - Cartoon illustration of expected MS2 and PP7 signals. (top) idealized pulse of constant initiation rate results in (bottom) trapezoidal MS2 and PP7 signals. If the promoter shuts off (black line) before the first cleavage process occurs (brown line), then the MS2 signal will plateau before the PP7 signal due to the former being located on the 5' end of the reporter gene.

2. During curation, roughly half of the single cell data that passed technical requirements are filtered and discarded, leaving a subset of trajectories that can be well-fit by the model. It should be reported what fraction of filtered cells are low signal:noise (S3E) and the fraction trajectories with poor fits (S3F). If the poor fits are significant in proportion, does this class of trajectories have any common features? An unfortunate requirement of standard Bayesian inference is that a model topology is itself assumed true. In combination with comment 1, it seems likely that the model, or its underlying assumptions, may be inadequate to reflect true biological complexity (which is probably always the case), but this has several consequences – one of which is that biophysical inferences may reflect a combination of biological rates associated with multiple cellular processes. The possibility exists that cells filtered because of poor fit indicate other states of the system that are not recapitulated in the model topology, but are reflected in another topology. Some of these limitations and ramifications associated with Bayesian inference would make relevant discussion at the discretion of the authors/editor.

Author response: In response to this and to the statements made by Reviewer 4's Point #12, we have overhauled our curation procedure to be completely automated.

To improve our dataset filtering procedure, we decided to remove the human element (which correctly was pointed out by several reviewers as non-rigorous and possibly bias-inducing) and implement an automated procedure.

The process now has two steps. First, we initially discarded any single cell time trace that did not have at least 30 time points in each fluorescent channel (the previous value was 10 timepoints). Over the 18 minute window of data acquisition at a time resolution of 15 seconds, this threshold corresponds to roughly half of the time window possessing detectable signal. We reasoned that traces with fewer than 30 time points would have an insufficient amount of data for the inference to work successfully. This reduced the number of cells from 1053 to 427.

Second, instead of manually curating the subsequent data and potentially introducing human bias, we opted for a new methodology that used an automatic cutoff. For each single-cell fit, we calculated the average squared normalized residual δ^2, defined as $\delta^2 = \sum_{timepoints}(F_{data} - F_{fit})^2/F_{data}{}^2$, where the summation occurs over all time points and $F_{data}$and F_{fit}correspond to the fluorescence data and fit, respectively. Thus, δ^2gives a measure of how good or bad, on average, each single-cell fit is.

Fig. R6A and B show histograms of the average squared normalized residual $\delta^2$for the entire n=427 dataset, with log and linear x-axes. We see that the vast majority of data possesses values of δ^2smaller than unity, with a long tail at higher values corresponding to bad fits. We decided to implement a cutoff of $\delta^2 = 1$, where any cell with a higher value of $\delta^2$was automatically discarded. This reduced the dataset from 427 cells to 355 cells (in the previous version of the manuscript size, the final dataset size was n=299).

To assess the rejected fits for underlying biological causes, we did a qualitative examination for common features. There were several sources of bad fits. First, some traces possessed low signal-to-noise ratio (Fig. R6C), possibly due to fluctuations in MCP-mCherry or PCP-GFP background fluorescences leading to increased uncertainty, that nevertheless yielded reasonable fits that were slightly above the δ^2cutoff. Still others simply had poor fits, possibly due to running into issues with the inference algorithm such as getting trapped in local minima (Fig. R6D). We consider improvements to the algorithm to be outside the scope of this work, since the retained data still contain novel, interpretable results.

Finally, one potential biological source confounding the model could be substantial burstiness of the promoter. Although the majority of the traces we analyzed indicated that the *hunchback* reporter gene studied here possessed a promoter that was effectively ON during the cell cycle studied, some traces possessed substantial time dependence of the fluorescence signal, potentially resulting from rapid switching of the promoter between ON and OFF states. From the lens of the model, this would violate

the mean-field assumption of the initiation rate term $R(t)$and cause the fluctuations $\delta R$to no longer be small compared to the mean value $< R >$. As seen in a representative example in Fig. R6E, such traces are very time-dependent and are not fit well with the model. Although such bursty behavior is of high biological significance, capturing the behavior would require more specific models (e.g. two-state telegraph models in the flavor of Lammers *et. al.* 2020 *PNAS*), and thus we hope to consider these extensions in future work.

Due to the variety of sources contributing to the rejected fits, we opted for a conservative approach and only analyze the cells with high signal quality that did not exhibit noticeable bursting. The number of retained fits were still much higher than the number of rejected fits (Fig. R6F). Thus, our work provides a self-contained framework applicable for describing the behavior of promoters that are primarily ON for the duration of the experiment.

To check that the curation procedure did not incur substantial bias, we compared the average inferred mean initiation rate, elongation rate, and cleavage time as a function of embryo position between the curated and uncurated datasets (Fig. R6G-I). We observed no substantial difference between the two datasets, indicating that the curation procedure was not systematically altering the inference results.

These details have been included in the updated Section S4.3 and the new Figure S4. We have also expanded the discussion to talk about these bursty traces, starting at Line 520.

3. The authors perform a nice control experiment treating their movies as snapshot data to infer sources of correlated and uncorrelated noise. Because CVs of their inference approach are comparable with the biological noise component from the snapshot analysis, the authors conclude that inference filters experimental noise and effectively captures biological variability only. It's not fully clear what criteria are used to establish that inference is capturing only biological variability only in Figs 2D and S7, and not some combination of variability with noise sources? Although it may be true that inference is filtering poisson noise, I can imagine other systematic sources of noise that would almost certainly be reflected in inferred parameters that do not reflect variation in a transcriptional parameters. For example, compare parameters inferred for a trajectory and for the same trajectory convolved with the line y = at+b which may represent a time varying fluctuation in the fluorescent coat protein – this type of noise will not be filtered by Bayesian inference. In addition, for this assertion to be true, the snapshot analyses must be limited to only the same curated cells that were analysed by inference (i.e. the ~30% of cells that passed the stringent filters), although it's not clear that this is the case.

Author response: We agree with the reviewer that our language was imprecise. Our method can only separate between uncorrelated and correlated sources of variability, which we imprecisely interpreted as experimental and biological variability, respectively. We have now amended our language in the main text and Section S8 to state that our inference method filters out the bulk of the uncorrelated variability and retains the correlated variability, which includes the biological variability. We have also clarified the text in Section S8 starting at Line 1369 to state that the snapshot analysis was limited to the same curated cells analysed by inference.

4. Although the writing and presentation is generally very clear, the results section seems awfully long and wordy, and requires regular flipping between the MS and supplement. For example, much of Figure 2 establishes that rates from inference are consistent with previous observations, yet the description is spread over 6 pages of text that require significant detail from various subsections of the supplement. A constructive criticism is to distill the results section, moving text as necessary to the intro/discussion, and recover some of the details from the supplement into the main paper. In my opinion, Figure S2 is an important representation of the fundamental approach that would be appreciated as a main figure by the computational readership, and there may be others at the discretion of the authors/editor.

Author response: We completely agree with the reviewer. To improve the clarity and flow of presentation, we have added two new figures to the main text with corresponding text and have rewritten parts of the introduction and discussion.We hope

that the reviewer will agree with us that the main text now reads more clearly and will require less consultation of the supplement.

Minor Comments:

1. The abstract builds the expectation that there is significant novelty in the computational algorithm, but the core approach is standard Bayesian inference with a few optimizations for these particular data. At the authors' discretion, I'd suggest edits to the abstract and introduction as necessary to keep these expectations realistic.

Author response: We agree with these suggestions and have edited the abstract, introduction, and discussion to more precisely state the novelty of our work. Namely, the computational algorithm itself is quite standard, but the application of Bayesian inference to directly fitting live imaging datasets is novel.

2. S3.3 curation suggests 260 cells were skipped from 1053 total leaving 567: these numbers don't add up.

Author response: After implementing a new automated curation procedure (see above response to Major Comment #2), these numbers have been updated.

3. Explanation/citation for the ranges of prior distributions in the supplement

Author response: We have added a paragraph with explanations and citations for the ranges of prior distributions in Section S3.1.

4. Full model seems to assume instantaneous and complete binding of fluorescent protein-fused PCP/MCP to stem loops. The assumption should be explicit and possible caveats discussed in the relevant section of the supplement.

Author response: We have clarified the text in the supplement (Section S1) to be clear about this instantaneous and complete binding assumption (starting at Line 969), and have also added text discussing the implications of this assumption (starting at Line 971).

5. More details are necessary to understand why simulated trajectories have poor S:N (line 931).

Author response: Previously, we classified some simulated trajectories as having poor signal to noise due to the incorporation of Gaussian fluorescence noise, using that as a basis for rejecting fits of certain simulated cells. Now, we have decided to forgo any

curation of simulated results and instead are running our analysis on the full simulated dataset, to prevent human bias. As a result, the point about poor signal to noise has been removed from the manuscript.

6. The discussion could benefit from description of how the experimental limitations (15s/frame) and inference limitations may be improved in the future. Also whether inferred parameters are likely to change significantly with more technological advancements.

Author response: We have added text in the Discussion starting at Line 509 discussing the impacts of experimental and inference limitations. To summarize briefly, while increased experimental and inferential resolution will likely sharpen single-cell results and reduce error, we do not believe that our results will change dramatically, since our current conclusions are already within statistical error. Furthermore, the model itself is coarse-grained enough where finer resolution will probably access regimes where the model will no longer apply (e.g. single-molecule resolution accessing fluctuations due to stochasticity in single molecule dynamics), requiring advances in modeling as well.

Reviewer #2:

The authors combine quantitative experiments of nascent transcription with computational modeling to simultaneously study the relationship between critical steps in transcription: initiation, elongation, and cleavage on nascent RNA. Studying these three processes together rather than independently, as often done in the literature, is an essential step toward understanding the transcription cycle. Integration of experiments with computational modeling is vital in this process.

I am overall enthusiastic about the problem and the importance of integration of modeling with experiments. I am also excited about the fact that the experiments have been performed in a multicellular organism. However, I have major reservations about why a model is necessary in the first place? What is the utility of the model besides data analysis? I also have concerns about the implementation and choice of modeling tools. Besides, I have concerns about the lack of cited references related to the same and competing modeling approaches from other groups. Moreover, I have reservations about the presentation of the results. Lastly, I have concerns about the lack of controls.

Author response: We thank the reviewer for their assessment. We have responded to their comments below, and hope that the reviewer will agree with us that the manuscript has been greatly improved after incorporating their suggestions and critiques presented.

Here are my specific concerns:

1. The model is not used to make any predictions for new or non analyzed data. Although I see the value in using a model to quantify the single-cell data, I have concerns about how predictive such a model is. How do the results change if one would use a piecewise linear fit to each of the sections of the single cell transcription time trace?

Author response: The primary purpose of our model is not to generate predictions on biological data, but rather to provide a first-principles framework to quantify effective transcriptional parameters that can then be used in predictive models. We now make this point more explicitly in the manuscript at Line 72. Indeed, we envision our methodology to provide a bridge between theoretical and experimental works. For example, a truly predictive biophysical model may involve parameters such as the elongation rate. These parameters are not easily or directly identifiable from raw microscopy data. Our work provides the middle ground that processes the raw data to generate the more biophysically motivated parameters that other models rely on.

This type of modeling approach is important because models not rooted in first principles, such as the piecewise linear fit posed by the reviewer, cannot easily be tied to underlying biophysical mechanisms. For example, a piecewise linear fit to fluorescence data inherently contains assumptions about what physical processes are generating the piecewise linear functional form. In this case, that includes a constant rate of initiation in order to produce a linear increase in fluorescence. Our first principles model explicitly writes down these biophysical processes in order to generate fluorescence predictions that arise directly from biophysical parameters, rather than empirical or heuristic fits.

Finally, while our model does not generate predictions *a priori*, it does provide fertile ground for posing new experiments. For example, our observed correlation between mean RNAP densities and cleavage times posits a quantitative relationship that has not been measured with current technology, providing a motivation for developing new experiments—and theory—that could measure and explain such relationships.

We have updated the Introduction to be more explicit about this type of reasoning.

2. A discussion should be included why there are differences in the distribution shape between the inferred single-cell parameters from the model fit and the distributions from the simulations (e.g. Fig2F). I believe the simulations make assumptions about the shape of the parameter distribution that differs from the data.

Author response: We agree that the previous analysis of distributions could have been more rigorous. In the new manuscript, we have updated the single-molecule simulations of elongation rates to provide a more meaningful comparison with the data by now simulating actual MS2 and PP7 fluorescences that are pushed through the inference pipeline instead of just calculating simulated elongation rates. The inferred elongation rate results from the simulation can then be more directly and sensibly compared to the empirical inferred distribution of elongation rates (Fig. 4D in the new manuscript).

With these new results, we now show that, while inferential noise widens the distribution of elongation rates in the absence of single-molecule variability in RNAP stepping rates, this distribution is still not wide enough to account for the observed data. In contrast, allowing for single-molecule variability can quantitatively recover the observed distribution within error, described in more depth in Section S10 and Fig. S10.

3. An explanation should be provided why R(t) is parameterized as a constant and a noise term. What are the advantages and disadvantages of this approach? The explanation should also include modeling approaches from other groups to understand the context of the work better.

Author response: Parameterizing R(t) using a mean-field approach with fluctuations captures a balance between reducing the number of free parameters in our model while also allowing for a slight time dependence. For example, completely relaxing R(t) to be an arbitrary time dependent vector underconstrains the model and will likely result in overfitting. Specifically, in this scenario we would expect the model to attempt to fit each nonlinear deviation in the fluorescence even though most of these deviations stem from measurement noise. On the other hand, the *hunchback* gene is known to possess time-dependent behavior (Garcia *et al.* 2013, *Current Biology*; Liu *et al.* 2013, *PLoS One*), slowly beginning to shut off its promoter over the time window studied here. Including the fluctuation terms allows the model to account for this time dependence.

This modeling decision should be considered complementary to other time-dependent models of initiation, for example the two-state telegraph (e.g., ON/OFF) model of bursty promoters (see, for example, Lammers *et al.* 2020, *PNAS*). While those classes of models attempt to explain genes that have bursty rates of initiation that are nevertheless well approximated by a binary set of ON/OFF states, here our gene appears to

overwhelmingly stay in an ON state with a more continuous interval of possible rates of initiation.

We have integrated a discussion of these caveats and assumptions into the introduction of the model starting at Line 107, and also into the Discussion at Line 520.

4. The authors stress in their abstract that the in-vivo dissection of initiation, elongation, and cleavage is challenging because of the lack of sufficient spatiotemporal resolution to separate the contributions from each of these steps. It is not clear in the context of the manuscript what the authors mean by that. Are you referring to the lack of spatiotemporal analysis of nascent, nuclear, and cytoplasmic RNA or referring to differences in expression within the organism? Both are important questions that could be answered in this model system. A differentiated discussion would be helpful for the manuscript, particularly in the context of the findings.

Author response: By spatiotemporal resolution, we mean spatial resolution within the body of an organism, and temporal resolution to resolve transcriptional processes in living cells on sub-minute time scales. While analysis of nuclear and cytoplasmic RNA can shed light on many biological processes, we have restricted this work to considering only nascent RNA labeling technologies in order to constrain the overall scope. The primary motivation for this consideration of spatiotemporal resolution is the fact that many transcriptional processes vary both in time and space, and that a nuanced analysis of the transcription cycle should possess the ability to parse this variation. In comparison with other technologies that require the use of fixed material such as RNA-FISH or GRO-SEQ, single-cell live imaging provides superior temporal resolution (compared to RNA-FISH) and superior spatial resolution (compared to GRO-SEQ).

We have updated the Discussion starting at Line 516 to clarify this point, and to mention the connections to analysis of nuclear and cytoplasmic RNA.

5. Also, in the abstract, the authors claim that the cell-to-cell variability is due to variability in the inferred parameters. It is not clear to me that this kind of statement is the only explanation. Alternative possibilities should be discussed that could contribute to these results.

Author response: Because our model invokes and infers effective transcriptional parameters, any cell-to-cell variability in transcription necessarily produces variability in the inferred parameters. This variability could stem from multiple sources, including experimental error, single-molecule stochasticity, or systematic variability in rates between cells, to name just a few examples. In this work we are not making definitive claims about the nature of these sources of variability, but rather providing a way to

quantitatively parameterize the transcription cycle to pave way for more first-principles, predictive models to tease apart these various sources.

We have updated the Introduction to be more clear about this point.

6. A discussion should also include the advantages and disadvantages of measurement and model inference from live cells compared to snapshots in time of single-cell measurements of transcription (RNA-FISH).

Author response: We have updated the Discussion starting at Line 440 to explicitly compare our live imaging methodology with fixed-tissue approaches such as RNA-FISH. Briefly, RNA-FISH provides superior spatial and molecular resolution at the expense of temporal resolution from within the same organism. Snapshots in time from different experiments using RNA-FISH are not equivalent due to the inherent variability between organisms and also the fixation process preventing high temporal resolution (e.g. ~15s as performed in this work).

7. The presentation of the data needs to improve substantially. Currently, many of the critical results are buried in the supplementary material. It is also unclear the intermediate steps from the concept to the final results as shown in Fig.2.

Author response: We agree with the reviewer and have restructured our manuscript to be more clear by bringing in some figures and text from the supplement. We have also modified the old Fig. 2 (now Fig. 4) to hopefully become more clear.

8. The author state that they measured 299 cells across 7 embryos. In the title and abstract, the authors state that they study cell to cell variability. So why are many of the plots showing population-averaged data? It would be much more insightful if the results are plotted as joint probability distributions for any of the graphs, instead of showing population averages, to appreciate the level of variability truly.

Author response: We respectfully disagree with the reviewer and think that our choice to present population means with standard errors in the old Fig. 2 (now Fig. 4) provides a more accessible presentation of the data. Particularly because the standard in the developmental biology community is to consider spatial variation of averaged data, our Fig. 4 provides easily interpreted results that many in the eukaryotic transcription community would find easily readable.

The latter half of Fig. 4, as well as Fig. 5, expand on this presentation to provide distributions, coefficients of variation, and correlation analysis. We believe that this provides a comprehensive summary of the data and that including the full probability distributions does not provide extra insight, especially in consideration of the fact that

the empirical distributions are confounded with inferential noise (Fig. S5 in the updated manuscript).

That said, full probability distributions provide a comprehensive way to visualize the data, and so we have included a new supplementary Section S7 and Fig. S7 that include these plots.

9. More of the supplementary figures should be moved to the main manuscript. I don't see any reason why the main manuscript needs to have only 3 figures.

Author response: We agree with the reviewer's assessment and have moved some figures to the main manuscript. Specifically, we have moved parts of Fig. S1, S2, and S5 into the main manuscript, along with the corresponding relevant text.

10. Interestingly, some of the parameters change within the embryo (lines 260-262). Unfortunately, the biological reason for this is not discussed, nor how the organism could regulate these changes. More discussion should be added to better present this exciting result.

Author response: We have added text in the Results section at Line 354 and in the Discussion at Line 430 emphasizing this result. To summarize, we speculate that the coupling of the cleavage time to embryo position could stem from processes such as promoter-terminator looping, where the cleavage time could couple to a position-dependent factor such as an activator.

11. Figure 1 outlines the different steps in the transcription cycle that can be inferred from the single-cell time-lapse data. Figure S1 should be integrated into Figure1. All the features that can be extracted from the single-cell time traces should also be included in this figure to make readers fully aware of the approach's power.

Author response: We have integrated Fig. S1 into Fig. 1 as recommended by the reviewer.

12. Besides the authors filtering out cells that do not have enough time points measured, the authors filter out 268 cells (567-299 retained after curation) that could not be inferred. This might bias their results if they filter data that their model can describe vs. improve/alter their model to describe those cells. The concern is that filtering might be removing some dynamics that could not be inferred by the model instead of just quality control. Experimental acquisition noise/Intrinsic Biological deviation from the model does not seem to me be enough justification for removing cells from the dataset. A comparison between the current data and the data with this additional curve should be compared to remove this does not change the results.

Author response: We agree that the curation procedure may be biased, and in response to this (as well as other reviewers' comments) we have overhauled the curation procedure to be fully automated, with no human input.

The process now has two steps. First, we initially discarded any single cell time trace that did not have at least 30 time points in each fluorescent channel (the previous value was 10 timepoints). Over the 18 minute window of data acquisition at a time resolution of 15 seconds, this threshold corresponds to roughly half of the time window possessing detectable signal. We reasoned that traces with fewer than 30 time points would have an insufficient amount of data for the inference to work successfully. This reduced the number of cells from 1053 to 427.

Second, instead of manually curating the subsequent data and potentially introducing human bias, we opted for a new methodology that used an automatic cutoff. For each single-cell fit, we calculated the average squared normalized residual δ^2, defined as $\delta^2 = \sum_{timepoints}(F_{data} - F_{fit})^2/F_{data}{}^2$, where the summation occurs over all time points and $F_{data}$and F_{fit}correspond to the fluorescence data and fit, respectively. Thus, δ^2gives a measure of how good or bad, on average, each single-cell fit is.

Fig. R6A and B show histograms of the average squared normalized residual $\delta^2$for the entire n=427 dataset, with log and linear x-axes. We see that the vast majority of data possesses values of δ^2smaller than unity, with a long tail at higher values corresponding to bad fits. We decided to implement a cutoff of $\delta^2 = 1$, where any cell with a higher value of $\delta^2$was automatically discarded. This reduced the dataset from 427 cells to 355 cells (in the previous version of the manuscript size, the final dataset size was n=299).

To assess the rejected fits for underlying biological causes, we did a qualitative examination for common features. There were several sources of bad fits. First, some traces possessed low signal-to-noise ratio (Fig. R6C), possibly due to fluctuations in MCP-mCherry or PCP-GFP background fluorescences leading to increased uncertainty, that nevertheless yielded reasonable fits that were slightly above the δ^2cutoff. Still others simply had poor fits, possibly due to running into issues with the inference

algorithm such as getting trapped in local minima (Fig. R6D). We consider improvements to the algorithm to be outside the scope of this work, since the retained data still contain novel, interpretable results.

Finally, one potential biological source confounding the model could be substantial burstiness of the promoter. Although the majority of the traces we analyzed indicated that the *hunchback* reporter gene studied here possessed a promoter that was effectively ON during the cell cycle studied, some traces possessed substantial time dependence of the fluorescence signal, potentially resulting from rapid switching of the promoter between ON and OFF states. From the lens of the model, this would violate the mean-field assumption of the initiation rate term $R(t)$and cause the fluctuations $\delta R$to no longer be small compared to the mean value $< R >$. As seen in a representative example in Fig. R6E, such traces are very time-dependent and are not fit well with the model. Although such bursty behavior is of high biological significance, capturing the behavior would require more specific models (e.g. two-state telegraph models in the flavor of Lammers *et. al.* 2020 *PNAS*), and thus we hope to consider these extensions in future work.

Due to the variety of sources contributing to the rejected fits, we opted for a conservative approach and only analyze the cells with high signal quality that did not exhibit noticeable bursting. The number of retained fits were still much higher than the number of rejected fits (Fig. R6F). Thus, our work provides a self-contained framework applicable for describing the behavior of promoters that are primarily ON for the duration of the experiment.

To check that the curation procedure did not incur substantial bias, we compared the average inferred mean initiation rate, elongation rate, and cleavage time as a function of embryo position between the curated and uncurated datasets (Fig. R6G-I). We observed no substantial difference between the two datasets, indicating that the curation procedure was not systematically altering the inference results.

These details have been included in the updated Section S4.3 and the new Figure S4. We have also expanded the discussion to talk about these bursty traces, starting at Line 520.

13. No experiments are performed testing if rates can be separated from each transcription cycle step. Potential further experiments would be to add inhibitors of regulatory proteins involved in nascent transcription initiation, elongation, and or cleavage to see if specific rates in the model associated with each transcription cycle change.

Author response: We have added text to the Discussion starting at Line 476 talking about the potential for future perturbative experiments to assess how rates in the model change as a result.

14. How does the insertion of the MS2 / PP7 repeat impact the transcription cycle? Comparing live-cell labeled nascent transcription with RNA-FISH on fixed cells with probes against MS2 or PP7 repeat is required to ensure that the repleads do not introduce artifacts.

Author response: Previous works utilizing MS2 and/or PP7 nascent RNA labeling technologies have shown by comparison with RNA-FISH that the insertion of these stem loops does not introduce substantial artifacts on transcription initiation (Garcia *et al.*, *Current Biology,* 2013; Coulon *et al., eLife,* 2014). More importantly, we do not believe RNA-FISH, or any existing technology for that matter, provides a concrete control to compare our experimental setup with, as there currently is no analogous technology for directly measuring the transcription cycle at the single-cell level and in live cells. A fixed-tissue technology such as RNA-FISH is unable to separate out the components of transcription initiation, elongation, and cleavage as done in this work, and thus would not be able to produce a direct comparison. Producing a framework to indirectly separate out these steps by assuming underlying model structures (e.g. Zoller *et al., Cell,* 2018) could constitute a feasible strategy, but such work would justify a new project in and of itself.

Because nascent RNA labeling technologies such as MS2 and PP7 have been widely adopted in recent years with many existing controls performed to confirm its robustness, we believe that carrying out an RNA-FISH experiment is unnecessary. While we agree that creating an orthogonal method to verify the results of our work would strengthen our confidence in our results, we hope that the reviewer will agree that the development of such new methodology lies outside the scope of this work.

Minor:

15. The statistical analysis in figure three was not the best suited for the data. The R^2 value is essentially meaningless due to the data not fitting a line, and linear regression, in general, is not ideal for data that is not normally distributed. I would suggest using a non-parametric test for correlation, such as the Spearman.

Author response: We have updated the analysis to use the Spearman non-parametric test for correlation instead of linear regression, and the main conclusions about RNAP density and cleavage times hold. We opted to retain the linear fits in Figure 3 (now Figure 5) for visualization purposes.

16. Inline 197, the authors state: "...convolved with undesired experimental noise.". What does this mean? Please provide more detail.

Author response: We have clarified this line by providing an example of undesired experimental noise, using the case of uncorrelated measurement noise from fluorescence microscopy measurements.

17. The manuscript's title is a summary of what has been done. But not the take-home message of the manuscript? I recommend rephrasing the title.

Author response: We have changed the title to "Real-time single-cell characterization of the eukaryotic transcription cycle reveals correlations between RNA initiation, elongation, and cleavage" and hope that the reviewer will agree that this new title more comprehensively summarizes the results of our work.

Reviewer #3:

the review uploaded as an attachment

Review for "Single-cell characterization of the eukaryotic transcription cycle using live imaging and statistical inference" by Liu et al.

Summary:

In this work, Liu et al. present a novel computational technique to simultaneously infer the effective parameters of the transcription cycle (including initiation, elongation, and cleavage of the nascent transcript) at the single-cell level from live-imaging of transcription using a two-color MS2/PP7 reporter gene. The authors apply this technique to study these parameters in developing fruit flies by analyzing the dynamics of ms2/pp7 gene controlled by the hunchback P2 promoter. From the fitted parameters' distributions, the authors show significant variability in the elongation rates between transcribing RNAP and a small negative correlation between the transcription initiation

rate and RNA cleavage time. The results are extensively compared with findings from previous works on transcription initiation, elongation and termination.

MS2 and PP7 reporter genes have been shown to be powerful tools to study the in vivo dynamics of transcription in many organisms, from bacteria to human. The combination of the two reporter systems is promising in revealing the parameters of transcription cycle, as demonstrated in the studies of e.g. elongation rates (Fukaya et al., 2017), RNA splicing kinetics (Coulon et al., 2014). Due to the fact that the fluorescence signals are usually from multiple nascent RNAs, a computational framework to extract the kinetic parameters of transcription cycle, as intended in this work, is welcomed.

Author response: We thank the reviewer for their assessment. We agree with their critique and have responded to their comments below. We hope the reviewer agrees with us that the new manuscript is greatly improved after incorporating their suggestions.

However, in this work, the model's assumptions are not adequately justified. The inference method, despite its flexibility to account for more complex models, is undermined by the arbitrary hierarchical fitting method. When applying the inference to individual traces, the systematic error is shown to be significant enough to affect the conclusions, especially regarding the parameters' variability and correlation.
As these issues are at the bottleneck of this work, it is difficult to evaluate the validity of the subsequent presentations and interpretations of the inference results from MS2/PP7 data, despite being well written in details.

Author response: We have overhauled the hierarchical fit procedure and have replaced it with a more suitable observation model. This new fit procedure did not exhibit the systematic errors in fitting earlier time points versus later time points that was shown in the old model. Please, see our response to point #8 below.

In addition, we would like to clarify that analysis on simulated data indicated that the systematic error in inferred values was negligible (Section S4.4 and Fig. S5). As a result, we hope that the reviewer will agree that the systematic error, though non-negligible, is not sufficient to obscure the correlations we have uncovered.

Major points:

1. As the conclusions are drawn entirely by fitting a 3-step model (line 92-107) to a single dataset of hunchback P2 reporter, the presented model needs to be justified first. Without this, it is difficult to conclude about the steps at the mechanistic level.

Author response: We intend our model to be less of a mechanistic explanation of the transcription cycle, but rather a simple parameterization of the key steps that provides *effective* values of the key steps—initiation, elongation, and cleavage. This reasoning is clarified more in the Introduction and Discussion. In addition, these 3-parameter models have been successfully used in prior studies of the same reporter gene (Garcia *et. al.* 2013 *Current Biology*, Eck *et. al.* 2020 *eLife*). While some other biological mechanisms are missing in our model (e.g. abortive initiation or nonprocessive RNAP molecules), we do not anticipate them being necessary for our reporter gene. For example, for our reporter we estimate that the vast majority of productively elongation RNAP molecules are processive (see Section S5 in the new manuscript) and the experimental readout can only resolve these productively elongating molecules, we do not anticipate factors such as incomplete elongation to play a role. Thus, we believe our model provides a sufficient parameterization of the relevant steps of the transcription cycle.

2. How the cleavage time can be distinguished from RNAP pausing at random or specific sites of the reporter gene (Herbert et al, Cell 2006)? Please clarify on the possible time scales of these steps to justify the preference of the cleavage time. I would also like to see how this technique can help in model selections.

Author response: Our experimental system should directly resolve the presence of substantial RNAP pausing within the main body of the reporter gene. For example, if RNAP molecules were to pause between the MS2 and PP7 stem loop sequences, then the corresponding fluorescent signal would exhibit delays or plateaus in the rising slope of the mCherry and/or GFP fluorescences. The cleavage time, on the other hand, entirely depends on how long the signal persists *after* the onset of the 3' GFP signal (see Fig. 1D in the new manuscript for a graphical explanation of this).

If pausing were to happen 3' of the GFP signal, then it is effectively indistinguishable from cleavage. However, we stress that our model is only an effective parameterization, and so we make no mechanistic claims as to the source of a particular cleavage time value. It could stem from pausing at the 3'UTR of the reporter, for example, or from continued elongation past the 3'UTR due to inefficient cleavage and termination processes. These would exhibit the same experimental signals—namely, persistence of fluorescent signal after the expected time of signal loss—and thus is a challenge of experimental resolution and not of model formulation. We now discuss this ambiguity in the Supplementary Section S1 starting at Line 982.

3. Transcription in eukaryotes, particularly in developing flies, has been shown to be very bursty. In Drosophila embryogenesis, the inferred ON-OFF periods are found to be ~ 1-5 minutes (Lammers et al. PNAS 2019, Desponds et al. PLOS CB 2016, Bothma et al. 2014), of the same order as the elongation time of MS2/PP7 transcript in this study ($\tau dwell$). Is this bursty dynamics accounted for in the temporal RNAP firing rate $R(t)$? If not, how does including bursty behavior affect the final conclusions?

Author response: The *hunchback* gene studied here primarily exists in an ON state in the time window analyzed, while slowly decaying to an OFF state over the course of the cell cycle. Because we have restricted our analysis to the first 18 minutes, where the gene remains mainly ON (Garcia *et al.*, *Current Biology,* 2013; Liu *et al.*, *PLoS One*, 2013), the system does not exhibit bursting, except in a small minority (4%) of cells that we have excluded from our analysis (see the updated Section S4.3 and Figure S4).

As it stands, our current parameterization of R(t) cannot accurately capture bursting because it does not assume a distinct set of ON-OFF states, but rather describes a continuous interval of initiation rates around some nonzero mean. As this manuscript presents a new approach for inferring values of live imaging data, we have restricted our analysis to promoters that are primarily ON, and consider bursty promoters to be outside the scope of this work. That said, bursting remains a biologically relevant feature of many systems and we believe future extensions to our work could benefit from integrating our approach in a hierarchical fashion with bursting models, such as the ON-OFF telegraph models referenced by the reviewer. We have added a discussion on this in the Discussion (Line 520).

4. Please clarify on the model: The fluctuation term $\delta R(t)$: is it free vector (arbitrary) term, correlated noise or uncorrelated noise? From the SI, it appears to be uncorrelated noise as its parameters are not inferred. Please clarify in the main text.

Author response: The fluctuation term is a deterministic free vector term that is constrained by imposing a Gaussian prior centered around zero (see Section S4.1 in the new manuscript). That is, at each time point of a trace the fluctuation term is independently inferred to be able to account for slight nonlinearities in the fluorescence, since the mean term will only produce a straight line in fluorescence. The presence of the Gaussian penalizes large fluctuations and exists to prevent overfitting to measurement noise. We have clarified the relevant text in Section S1 and S4.1 to include this information more explicitly.

5. If $\delta R(t)$ is uncorrelated noise, the fluorescent signal should be deterministic (as in Fig. 1). Why there are fluctuations in the prediction of the model (Fig. S3, Fig. S4) or the downward trend in Fig. 2B after 10 minutes.

Author response: These fluctuations exist because the fluctuations $\delta R(t)$ serve as independent constant offsets at each time point to introduce and account for slight time dependence in the overall initiation rate. Biologically, the *hunchback* gene slowly transitions from an (approximately) constant ON state to an OFF state. Our time window of analysis captures the start of this decay, hence the downward trend of fluorescence at the end of the time window. This point is clarified in the main text at Line 141.

6. Does $R(t)$ account for the promoter bursty dynamics (Desponds et al, PLOS CB, 2016) or only dynamics of initiation during the ON period (a single burst)? How would this bursty dynamics affect the fluorescent traces?

Author response: See our answer to the point above on bursting. The *hunchback* gene primarily stays in an ON state for this reporter construct. Bursty dynamics would cause the fluorescent traces to oscillate dramatically in time (see e.g. Lammers *et al.*, *PNAS,* 2020 and the new Fig. S4E). Our framework is only applicable for traces that are in an ON period, and we envision future extensions to this work to account for bursting. We have included this caveat in the Discussion starting at Line 520.

7. Do you assume Gaussian noise on top of the signal from gene loci?

Author response: Our model previously assumed constant Gaussian noise, as parameterized by the sum-of-squares function in the old Eq. S12 and S13. In response to a point raised by Reviewer #4, our model now assumes Gaussian noise with variance that scales linearly with the mean (see Section S4.2 in the new manuscript). This new observation model fits the data much better and obviates the need for the hierarchical fit procedure.

8. The hierarchical fitting method seems confusing and arbitrary. First, the authors show that the inference from some longer (18 minutes) traces does not fit well with its early time points (line 870). For these traces that are not well fitted, only shorter (10 minutes) traces are fed to the inference (line 875). Then, the authors show that the refitted model from the short traces can generate traces that capture the dynamics in longer traces (line 885). In principle, it is the model's fault if it could not explain the data. For example, the promoter can turn OFF, leading to changes in loci fluorescent intensity after the initial uphill slope. I think data treatment for all traces should be done BEFORE the fit, rather than after the fit and only to a subset of traces.

Author response: In response, to a point brought up by Reviewer #4, we have developed a new observation model for our inference procedure that obviates the problematic hierarchical fitting method, as pointed out by the reviewer. The new model does a much better job of fitting both early and late timepoints, and we have thus removed the hierarchical fitting method from the analysis in the new manuscript.

To develop this new observation model, we decided to investigate the nature of the fluorescence measurement noise in our data. *A priori*, if we consider that the fluorescent signals in our experiment are the result of the sum of many individual fluorophores, then we would expect that if an individual fluorophore possesses some intrinsic constant measurement error with variance σ^2, then the associated error of Nfluorophores would have a similarly scaled overall measurement error with variance $N\sigma^2$. Since $N$is proportional to the overall mean fluorescent signal, we thus hypothesize that our observation model would be improved by considering a scaled residual where the sum-of-squares error is divided by the signal intensity: $SS = (F_{fit} - F_{data})^2/F_{data}$, where $F_{fit}$and F_{data}correspond to the individual predicted or measured fluorescence intensities at each time point, respectively.

To check this approach, we examined the data from the dual-color interlaced MS2/PP7 reporter construct from Fig. 3B. These data constitute, in principle, a two-point measurement of the same underlying biological process, so we reasoned that we could utilize this measurement to quantify the scaling of fluorescence noise with respect to overall fluorescence intensity.

Specifically, by creating bins of eGFP fluorescence measurement from the scatterplot in Fig. 3D, we could then calculate how the variance of associated mCherry fluorescences within a bin scaled with eGFP fluorescence (here a proxy for overall fluorescence intensity). If the calculated variance increases with overall fluorescence, this indicates that the fluorescence measurement noise is not constant, but rather scaled positively with signal strength.

Fig. R3A shows this calculated variance (red), along with bootstrapped standard error, as a function of bin value (i.e. eGFP fluorescence). We see that the variance indeed increases with bin value fairly linearly, confirming our *a priori* hypothesis. If we then scale the variances by dividing by the mean mCherry fluorescence within a bin, we recover a constant scaling, as expected (black).

Thus, in the revised manuscript, we updated the observation model to include this scaled fluorescence measurement noise by dividing the sum-of-squares residual by the observed fluorescence intensities, as described by the equation above and in the updated Equation S14. We have also added a new section S4.2 describing this fluorescence noise scaling behavior.

Next, we investigated how this new observation model performed in terms of inference on the data. After implementing the modification to the observation model described in the previous point, the new inference procedure performs much better in terms of fitting earlier time points such that the hierarchical fit was no longer necessary. This is to be expected, since scaling the sum-of-squares residual causes lower intensity values (and thus earlier time points) to be weighted more strongly than higher intensity (i.e. later) values.

Fig. R4 shows data from a sample representative single-cell. In Fig. R4A, we used the old hierarchical fit with the previous observation model, which results in a discrepancy between the fit and data at earlier time points, exemplified by examining the onset of the GFP signal (green). In contrast, Fig. R4B shows the fit from using the new observation model with a scaled residual term, which fits the onset of GFP signal much better. Thus, the hierarchical fit was no longer necessary, and we decided to remove it. We believe the new methodology is much more statistically sound and hope the reviewer will agree. As a result, we have removed the old Section S3.2 describing this hierarchical fit procedure.

9. The model that fits the short traces is shown to capture the longer traces' dynamics, which were not well fitted before. Intuitively, this suggests a bias in the inference from individual traces, as demonstrated with simulated data.

Author response: Please see the above response. The new model behaves much better with fitting short and long traces.

10. When testing the inference on simulated data (Fig. S4C), the scaled error (or bias per cell) distribution is symmetric indicating that the estimation of the ensemble mean parameter values may be correct. However, the scaled error's ranges of -0.5 to 0.5 should be considered significant, as it is of the same order as the CV of the inferred parameter distribution (Fig. 2). Therefore, conclusions on the variability of the elongation rates should all be reconsidered.

Author response: We agree that the old conclusions on the variability of elongation rates could be made more rigorous. We have updated the single-molecule simulations of elongation rates to provide a more meaningful comparison with the data by now simulating actual MS2 and PP7 fluorescences that are pushed through the inference pipeline instead of just calculating simulated elongation rates. Because now both simulation and data are processed with the same inference procedure, confounding factors such as inferential noise will be present in both experimental and simulation analyses. Thus, the -0.5 to 0.5 scaled error is already manifested in both analyses, and does not need to be taken into account in downstream comparisons. The inferred elongation rate results from the simulation can then be more directly and sensibly compared to the empirical inferred distribution of elongation rates (Fig. 4D in the new manuscript).

With these new results, we now show that, while inferential noise widens the distribution of elongation rates in the absence of single-molecule variability in RNAP stepping rates, this distribution is still not wide enough to account for the observed data. In contrast, allowing for single-molecule variability can quantitatively recover the observed distribution within error, described in more depth in Section S10 and Fig. S10.

11. Does the scaled error reduce with more nuclei/embryo?

Author response: Because the scaled error is defined as the error due to inference for a single cell, it is an intensive quantity that is independent from the overall dataset size. So, increasing the number of cells, for example in the inference simulation used in Section S4.4 and Fig. S5 will merely reduce the uncertainty in overall measures of the scaled error, such as the error bars in the ensemble squared CV shown in Fig. S5D. This is clarified in Section S4.4 at Line 1230.

12. Regarding the MCMC inference method, for each cell, after extracting the posterior distribution of the parameters, why the mean value is retained, rather than the mode (best fit)?

Author response: Because we had no *a priori* justification or expectation for the shapes of the inferred posterior distributions, calculating the mode would be difficult

since we would need to estimate a continuous PDF from empirical data. Thus, we retained the mean value rather than an estimate of the mode.

Additionally, in response to Reviewer #4, we conducted a deeper exploration of the single-cell correlation analysis in Fig. 5 in the new manuscript by considering the full posterior distribution (Fig. S11 in the new manuscript), and found that the conclusions did not change. Given this reassurance, we think our choice of using the mean is justified and validated.

13. I would like to see more discussion on the findings (e.g. variability in the elongation rates, RNAP crowding at termination site) and which additional experiments (e.g. additional ms2/pp7 configurations) that can, in complement with this approach, validate these findings.

Author response: We have added text in the Discussion starting at Lines 458 and 476 exploring the ramifications of our discoveries and potential future experiments to conduct.

Minor:

14. Until reading the detailed model section of the Appendix, I had the impression that elongation times of ms2 and pp7 stemloops (~1kb?) are considered negligible. Please add the length in base pair of the ms2, pp7 stem loops, lacZ and lacY in Fig. 1C, or at least some text in the main text to clarify.

Author response: We have added the lengths of these sequences to Fig. 1C.

15. Figure 1D, panel iii, left: R should represent the height of the slope, not the duration.

Author response: We have moved <R> in the figure to correct this.

16. Figure 2B: it is not clear whether the data points are taken from discrete uniform time interval. When active loci is not detected, do you assign a zero intensity value or the background value?

Author response: Data points are taken from a discrete uniform time interval of 15 seconds, with short (~3 sec) gaps every so often due to the experimental need for manually adjusting the confocal stack of the microscope. The fluorescence at each time point is subtracted by the background value as a rule. When active loci are not detected, they are assigned an intensity value of NaN. We have clarified this in the Materials and Methods section.

17. Readers would benefit from Fig. S1C being moved to the main text as it provides intuitions on how each parameter can affect the final trace.

Author response: We have replaced the old Fig. 1D with Fig. S1C, which provides a more complete picture of the model's behavior.

18. 100 cells for the evaluation of the inference seems very small, given the number and variability in distribution of the input parameters.

Author response: We agree with the reviewer that 100 cells is a small number for statistical purposes. The simulated dataset has now been expanded to possess 300 cells, which is sufficient to compare statistics with the dataset. For example, in Fig. S5D, the simulated inference's error in scaled squared error is sufficiently small to be able to definitively conclude that inferential noise is reasonably small compared to overall empirical variability in the inferred transcriptional parameters.

Reviewer #4:

%%%% What are the main claims of the paper and how significant are they for the discipline?

The authors investigate transcription dynamics in live cells using the well-established technique of labelling nascent mRNA molecules using the MS2/PP7 system. The main contribution of the paper is a deterministic model that describes transcription in terms of a few fundamental kinetic parameters. Despite its simplicity, the calibrated model is able to predict measured fluorescence levels fairly well. This is demonstrated on experimental data from the hunchback gene in the Drosophila embryo, which is known to show spatial variability of transcription levels.

%%%% Are these claims novel? If not, which published articles weaken the claims of originality of this one?

1. In my opinion the authors make too strong claims regarding the novelty of their general approach of using inference techniques in single-cell studies. I am aware that statistically sound inference is rarely done in the physics community (where it is usually degraded to a fitting procedure) but there is ample of work from the statistics and machine learning community to develop dedicated and sound inference schemes for single-cell data (e.g. papers of Finkenstadt and Rand, D. Suter et al. Science 2011, Zechner et al. Nature Methods 2014 and many more).

Author response: We thank the reviewer for bringing these related works to our attention and have added new text in the discussion starting at Line 480 discussing our work in the context of this existing literature. We view our work as providing a much-needed framework for statistical analysis of single-cell live imaging transcriptional data, extending the repertoire of existing statistical techniques for single-cell biological datasets.

2. For parameter inference, a semi-Bayesian perspective is adopted. In particular, an individual Markov chain is run for each cell. The posterior samples are used to estimate the posterior mean per parameter per cell. Then, the authors use descriptive statistics on these MAP estimates. As MAP estimates are usually rather sensitive (in particular for sampling-based approaches) I am a bit concerned whether the weak correlations they are finding and interpreting are really significant. A more rigorous approach would have been to work with the full posterior distribution.

Author response: We opted to use descriptive statistics on the posterior mean for each parameter since we were primarily interested in investigating variability between cells, rather than distributions of inferred values within a single cell. Although investigation of higher moments has been successful in FISH studies (e.g., Zoller *et al.*, *Cell*, 2018), even mean analyses are still bearing fruitful insights in live imaging experiments. Thus, in this work we decided to focus on mean-level analysis and consider a detailed examination of the full posterior for future works. Thus, in the main text we present single-cell correlations based on posterior means.

Nevertheless, we decided to ensure that these analyses were not biased. To do so, we extracted the mean and variance of each inferred parameter from the posterior distribution. We then conducted a Monte Carlo simulation to simulate a distribution of Spearman correlation coefficients (which we now use instead of Pearson correlation coefficients at the suggestion of Reviewer #2’s Point #15) and associated p-values to gain a sense of how accurate the results of the single-cell correlation analysis were. Using the mean and variance of each inferred parameter, we simulated N=50,000 new values of the mean initiation rate, elongation rate, and cleavage time, where these values were generated from Gaussian distributions parameterized by the means and

variances from each parameter's posterior distribution. We then calculated an individual Spearman correlation coefficient and associated p-value for each simulation, generating a distribution for each correlation relationship.

Fig. R2A and B show the ensuing distribution of p-values for the Spearman correlation coefficient between the mean initiation rate and elongation rate, as well as between the elongation rate and cleavage time. The p-values for the relationships between the mean initiation rate and cleavage time and between the mean RNAP density and cleavage time were essentially zero due to floating point error. Finally, Fig. R2C shows the simulated distributions of Spearman correlation coefficients for all four relationships (histograms), along with the values obtained from the simpler mean analysis presented in the main text (dashed lines). We see that using the full posterior via this Monte Carlo simulation yields distributions that are in agreement with the results from the mean analysis, and that the distributions themselves are fairly narrow. Thus, our original analysis is robust, and we chose to retain its presentation in the main text for simplicity and ease of understanding. This new analysis using the full posterior is included as a new supplementary section S11.

In addition, our investigation of simulated data provide a window into possible systematic bias in the inferred means (Fig. S5). Because the single-cell correlation analysis shows correlations in the data that do not exist in the simulated data, we believe that our results are not merely the product of inferential artifacts.

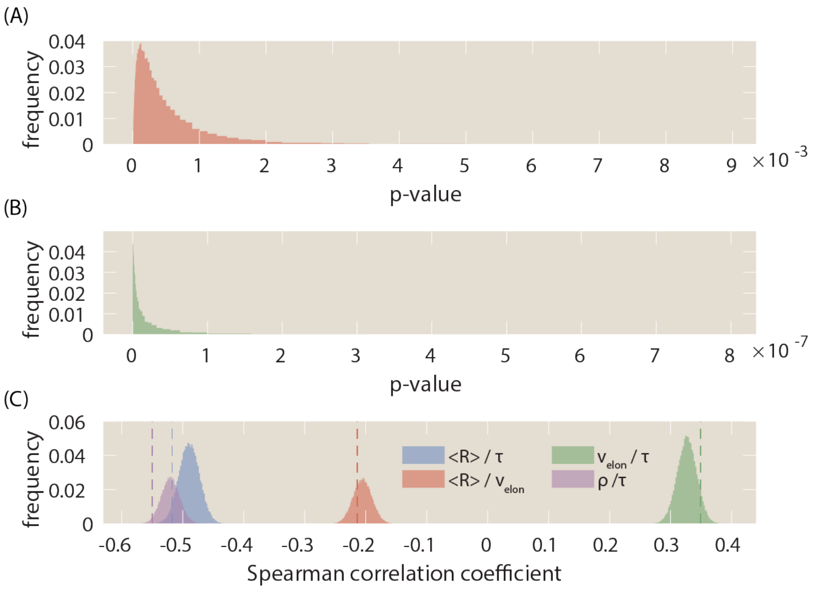

Figure R2 - Monte Carlo simulation of error in single-cell analysis. (A, B) p-values of Spearman correlation coefficient for relationships between (A) mean initiation rate and elongation rate and (B) between elongation rate and cleavage time. The p-values for the relationships between mean initiation rate and cleavage time as well as between mean RNAP density and cleavage time were essentially zero

due to floating point error. (C) Distributions of Spearman correlation coefficients between mean initiation rate and cleavage time (blue), mean initiation rate and elongation rate (red), elongation rate and cleavage time (green), and mean RNAP density and cleavage time (purple). Results from mean-level analysis are shown in dashed lines.

3. From a computational perspective, the Monte Carlo inference procedure is quite standard. Considering this, the promise of a “novel computational technique to simultaneously infer […] parameters” as mentioned in the abstract may be a bit over the top.

Author response: We agree with this point, as other reviewers have also pointed this out. We have adjusted our language throughout the abstract, introduction, and discussion accordingly. The computational technique is no longer regarded as novel, and we rather emphasize the novelty of applying a well-known statistical inference technique like Markov Chain Monte Carlo for the analysis of live imaging fluorescence microscopy datasets.

4. Nevertheless, the demonstrated results such as the spatial variation of the initiation rate are interesting. In my opinion, the most compelling methodological result is the use of the dual reporter system to eliminate the need for GFP calibration experiments. This is important, since GFP calibration has been a major drawback for model-based approaches in this area so far.

Author response: We appreciate the reviewer’s assessment of the usefulness of our dual reporter system. To emphasize its importance, we have moved the SI section on this calibration experiment to the main text, as well as the SI figure to become the new Fig. 3 in the main text.

%%%% Are the claims properly placed in the context of the previous literature? Have the authors treated the literature fairly?

5. Related work is not fully captured (see above) although the list of references is rather extensive with respect to nascent mRNA labeling work. What I found surprising though, is that auto-correlation analysis (e.g. applied by Larson and co-workers) was not discussed at all. To my knowledge, this is a standard technique to analyze live cell transcription traces. I would have expected a discussion of the advantages and disadvantages of the proposed method compared to ACA.

Author response: This is a pertinent comparison to make, and we have now included a discussion on advantages and disadvantages of our technique compared to auto-correlation analysis in the discussion, at Line 480. To summarize, we view auto-correlation analysis as a complementary method to our own, with its own set of strengths and weaknesses depending on various factors.

First, auto-correlation analysis typically requires a time-homogeneous transcript initiation process (Coulon and Larson 2016, *Methods in Enzymology*), and benefits immensely from having experimental data acquired over long time windows to enhance the auto-correlation signal. In contrast, our model-driven inference approach can account for slight time dependence and directly fits to time traces. This is of particular relevance to the fly embryo, where each cell cycle in early development is incredibly short (here, we only examined 18 minutes of data) and transcription initiation switches from OFF to ON and back to OFF within that time frame. As a point of comparison, Coulon *et al.*, *eLife* (2014) very successfully used auto-correlation analysis to examine splicing in human cells, but here the data were acquired over hundreds of minutes with a more time-homogeneous gene.

Secondly, auto-correlation analysis depends strongly on signal-to-noise ratio, namely the ability to resolve single-or-few-transcript fluctuations in the number of actively transcribing polymerases on a gene. This can be achieved via several methods, such as having single-polymerase resolution (Larson *et al.*, *Science, 2011*) or utilizing intronic splicing to produce fast, observable fluctuations in the signal (Coulon *et al.*, *eLife*, 2014). In our system, we do not have observable splicing and our signal-to-noise ratio is poor enough to only be able to resolve differences in transcript number of several transcripts, rather than just one.

Finally, our model-driven approach benefits from explicitly parameterizing the various steps of the transcription cycle, allowing for the separation of processes such as elongation and cleavage. In contrast, while the auto-correlation technique has the advantage of not relying on a particular specific model, it does rely on unknown parameters such as the overall transcript dwell time, which is a combination of elongation and cleavage. Thus, it becomes harder to separate contributions from these different processes. Furthermore, the overall auto-correlation signal benefits greatly

from averaging the auto-correlation signals from many individual cells. If there is large cell-to-cell variability in the transcription cycle, causing, for example, variability in the single-cell dwell time, then this averaging method becomes less useful. In contrast, our method reports on single-cell behavior and only needs averaging to produce summary statistics.

%%%% Do the data and analyses fully support the claims? If not, what other evidence is required?

6. The proposed inference scheme relies on an established Monte Carlo procedure and seems to work fairly well. However, it may be helpful to refine the observation model given in (S12), (S13). The given likelihood function implicitly assumes that given the true intensity, the observations are distributed with a standard deviation of one around the true value. Such a small observation noise is not realistic for fluorescence measurements. I would propose to consider a multiplicative noise model (larger noise for larger intensity) or at least some relative error in (S13). This may also help with the problem that the tails gets too much emphasis during the fit as discussed in S3.2.

Author response: We agree that assuming a constant fluorescence measurement noise is unrealistic, and decided to investigate this line further to figure out a more precise observation model. *A priori*, if we consider that the fluorescent signals in our experiment are the result of the sum of many individual fluorophores, then we would expect that if an individual fluorophore possesses some intrinsic constant measurement error with variance σ^2, then the associated error of Nfluorophores would have a similarly scaled overall measurement error with variance $N\sigma^2$. Since $N$is proportional to the overall mean fluorescent signal, we thus hypothesize that our observation model would be improved by considering a scaled residual where the sum-of-squares error is divided by the signal intensity: $SS = (F_{fit} - F_{data})^2/F_{data}$, where $F_{fit}$and F_{data}correspond to the individual predicted or measured fluorescence intensities at each time point, respectively.

To check this approach, we examined the data from the dual-color interlaced MS2/PP7 reporter construct from Fig. 3B. These data constitute, in principle, a two-point measurement of the same underlying biological process, so we reasoned that we could utilize this measurement to quantify the scaling of fluorescence noise with respect to overall fluorescence intensity.

Specifically, by creating bins of eGFP fluorescence measurement from the scatterplot in Fig. 3D, we could then calculate how the variance of associated mCherry fluorescences

within a bin scaled with eGFP fluorescence (here a proxy for overall fluorescence intensity). If the calculated variance increases with overall fluorescence, this indicates that the fluorescence measurement noise is not constant, but rather scaled positively with signal strength.

Fig. R3A shows this calculated variance (red), along with bootstrapped standard error, as a function of bin value (i.e. eGFP fluorescence). We see that the variance indeed increases with bin value fairly linearly, confirming our *a priori* hypothesis. If we then scale the variances by dividing by the mean mCherry fluorescence within a bin, we recover a constant scaling, as expected (black).

Thus, in the revised manuscript, we updated the observation model to include this scaled fluorescence measurement noise by dividing the sum-of-squares residual by the observed fluorescence intensities, as described by the equation above and in the updated Equation S14. We have also added a new section S4.2 describing this fluorescence noise scaling behavior.

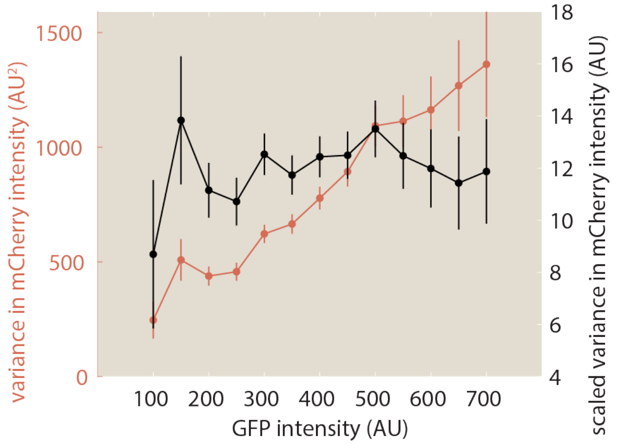

Figure R3 - Scaling of fluorescence measurement noise with overall fluorescence intensity. Variance of mCherry fluorescence at a particular GFP fluorescence (red), from the dual-color interlaced reporter construct from Fig. 3B, along with variance scaled by dividing out the mean mCherry fluorescence (black).

7. In my opinion, the hierarchical procedure proposed in S3.2 is a bit of a hack. There are more transparent methods to solve this problem, such as alternative observation models or using weighted residuals. The motivation to call some data points less important always seemed to be rooted in the bad fit they generate if considered full. A more sound way to model such discounting is through introducing heteroscedasticity in the observation model but that then still requires biophysical justification.

Author response: We agree that the hierarchical procedure is indeed a hack. We appreciate the reviewer for pointing out the necessity (and subsequent utility) of using

an improved observation model above. After implementing the modification to the observation model described in the previous point, the new inference procedure performs much better in terms of fitting earlier time points such that the hierarchical fit was no longer necessary. This is to be expected, since scaling the sum-of-squares residual causes lower intensity values (and thus earlier time points) to be weighted more strongly than higher intensity (i.e. later) values.

Fig. R4 shows data from a sample representative single-cell. In Fig. R4A, we used the old hierarchical fit with the previous observation model, which results in a discrepancy between the fit and data at earlier time points, exemplified by examining the onset of the GFP signal (green). In contrast, Fig. R4B shows the fit from using the new observation model with a scaled residual term, which fits the onset of GFP signal much better. Thus, the hierarchical fit was no longer necessary, and we decided to remove it. We believe the new methodology is much more statistically sound and hope the reviewer will agree. As a result, we have removed the old Section S3.2 describing this hierarchical fit procedure.

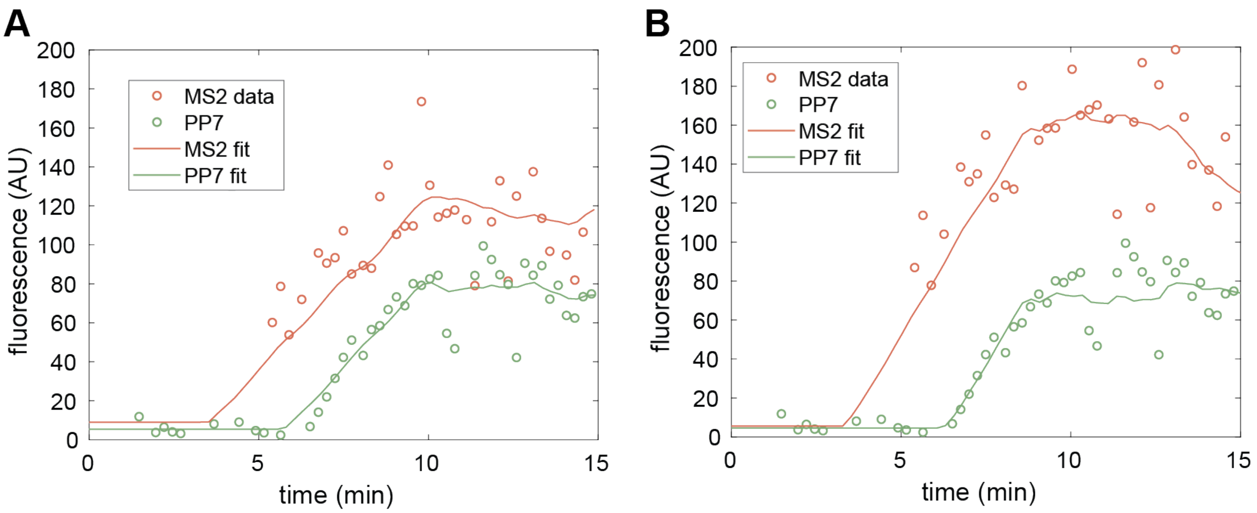

Figure R4 - Comparison of hierarchical fit methodology with improved observation model. (A) Fit results using old hierarchical fit with constant fluorescence noise observation model. (B) Fit results using improved, scaled fluorescence noise observation model, obviating the need for a hierarchical fit. The mCherry fluorescence values between methodologies is different due to a different calibration factor being inferred between models.

8. Testing the inference procedure on simulated results (S3.4) is helpful. I am surprised, though, that the inference result is so biased (Fig. S4 a). Considering that the model is relatively simple and the number of data points is large, I would not have expected such a mismatch. For me, this indicates some problem with the inference procedure. For example, the posterior could be multi-modal and the chain could be trapped in a local mode. The normalized error measure does not help to resolve this discrepancy.

Author response: With the new observation model, the inference has been much improved. As seen in the new Fig. S5A, the model fits well to the simulated data, with only slight deviations due to the addition of simulated fluorescence noise.

9. In my opinion, more evidence is required for section S4. This part is based on a construct with 24 alternating MS2-PP7 stem-loops. This model has been established first using sm-FISH using FISH probes (Zoller et al.,2018). Compared with the MS2-PP7 system, the FISH probes can be labeled with a lot of fluorophores, so it is more convenient to measure fluorescence intensity. By using the MS2-PP7 system, normally more than 14 consecutive stem-loops should be used to generate a single spot. It would be helpful to provide more figures or videos in this section. Also, the photobleaching needs to be considered by using this system. It would be better to mention more details about the DNA construct in this part as well. The measurement condition should be mentioned in figure 5S.

Author response: To clarify, the alternated MS2-PP7 construct has a total of 48 stem loops (24 of each type of stem loop), so the overall signal is definitely bright enough and over the 14-stem loop threshold suggested by the reviewer. We have updated the text accordingly to make this clear, as well as updated Figure S5 (now Fig. 3 in the main text) with the measurement conditions. We've additionally uploaded a new supplementary video S2 showing the live cell microscopy of this experiment—-both MS2 and PP7 signals exhibit similar dynamics and reflect the same underlying biological signal.

To check for photobleaching, we conducted an experiment with the dual-color 5'/3' tagged reporter where half of the field of view was illuminated using the experimental settings described in the Methods and Materials section (Fig. R5A, purple), and the other half was illuminated at half the temporal sampling rate (Fig. R5A, yellow). Since the measurement conditions were identical for both reporter constructs used in this work, the bleaching behavior (if any) should be the same. Thus, if the experimental settings were in the photobleaching regime, then the purple region would exhibit fluorescence at a systematically lower intensity compared to the yellow region.

Fig. R5B, C shows the fluorescence intensities of mCherry and eGFP as a function of time at a particular anterior-posterior position of the embryo for both 0.5x and 1x

sampling rates, where data points indicate fluorescence averaged within the anterior-posterior position (i.e. vertically in the field of view) and error bars indicate standard error across cells. There is no obvious systematic difference between the differentially illuminated regions.

To quantify this more accurately, we calculated the average normalized difference between illuminated regions, obtained by subtracting the fluorescence value at 1x sampling rate by that at 0.5x sampling rate, dividing by the fluorescence value at 0.5x sampling rate, and then averaging across all time points and embryo positions. For example, for the curves shown in Fig. R5B, this entails subtracting the red curve by the black curve, and then dividing by the black curve—and then averaging for all anterior-posterior embryo positions. An overall value of less than one means that the 1x sampling rate produces systematically lower fluorescence intensities, indicating that our experimental settings are in the photobleaching regime. As seen in Fig. R5D, the average normalized difference is around one for both fluorophores (within standard error, measured across all time points and anterior-posterior positions). Thus, we conclude that our data are not in the photobleaching regime.

We have added this information on photobleaching as a new supplementary Section S2 and Fig. S2.

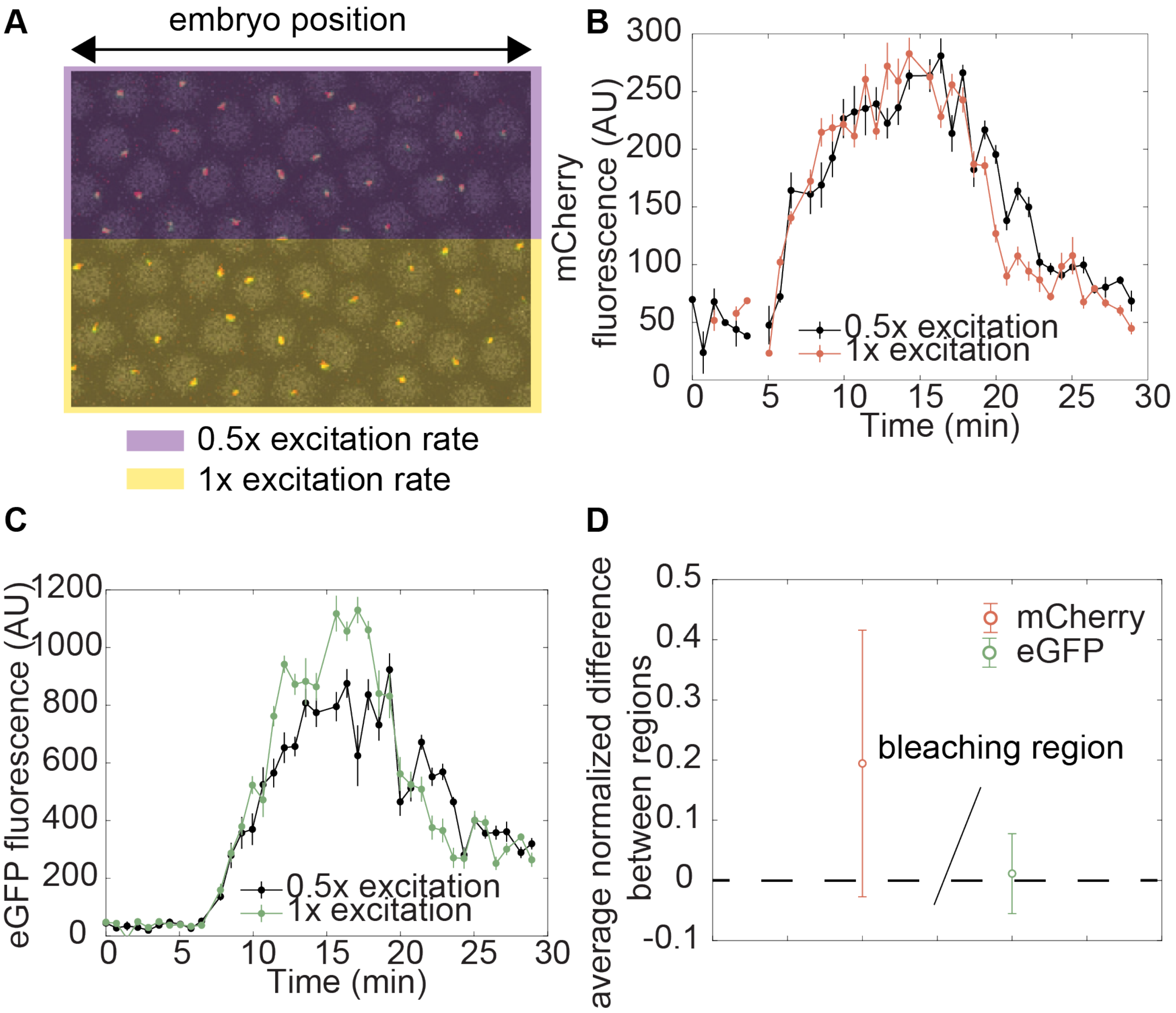

Figure R5 - Investigation of photobleaching. (A) Control experiment where half of the field of view is illuminated at the standard experimental settings (yellow), and the other half of the field of view is imaged at half of the illumination rate (yellow). (B, C) The (B) mCherry and (C) eGFP fluorescence signals at a given anterior-posterior embryo position, averaged across cells within that position, do not exhibit photobleaching. (D) The average normalized difference between illuminated regions, averaged across time points and anterior-posterior embryo positions, are approximately zero within error, rather than negative, which would indicate photobleaching.

10. The most problematic aspect of the paper seems to be the simulation study in S9. The idea is to support the claim of individual RNAPs having different step sizes by consulting a more elaborate simulation model of the transcription process. I see, however, some severe problems with the taken approach. First, the simulation itself is not very meaningful. Essentially, the result is that randomized RNAP step sizes produce random elongation rates, which seem quite trivial. Second, the authors compare this distribution of elongation rates to the distribution of inferred elongation rates. Conceptually, it does not make much sense to compare the distribution of inferred quantities with the distribution of a completely different generative process. What the authors could have done instead is to extend the fine-grained model in such a way that it can produce artificial observations. The artificial observation could then be used for inference as in S3.4. The obtained distribution of posterior means would allow an appropriate comparison with the distribution of inferred elongation rates from the real data.

Author response: We agree with the reviewer's critique of our approach. In response, we have updated the single-molecule simulation analysis of elongation rates to produce simulated MS2 and PP7 fluorescences that are then pushed through the inference pipeline, to produce results that can be directly and sensibly compared to the empirical inferred distribution of elongation rates. While inferential noise widens the distribution of elongation rates in the absence of single-molecule variability in RNAP stepping rates, this distribution is still not wide enough to account for the observed data. In contrast, allowing for single-molecule variability can recover the observed distribution.

We believe this result is not trivial. For example, due to effects such as stochasticity in RNAP stepping rates as well as traffic jamming due to steric hindrance, cell-to-cell variability could presumably manifest as an emergent phenomenon even in the absence of real single-molecule variability. Our simulation results, while far from conclusive, do provide preliminary exploratory work suggesting that the empirical cell-to-cell variability is large enough that these emergent phenomena are insufficient to reproduce them.

11. The filtering of data-points for the synthetic data scenario is beyond justification. If the data was generated according to the model that is later used inference, every datapoint needs to be taken into account.

Author response: We agree, and have updated the simulation validation (Section S4.4) to reflect this. We no longer filter out any of the synthetic data points and instead analyze the whole population.

12. The aggressive down selection performed on the real dataset appears also very problematic. From the original 1053 cells after successive filtering only 299 remain in the inference dataset. For some data points, the only justification to discard them is that they cannot be well explained by model. In my opinion that is an elementary statistical fallacy.

Author response: To improve our dataset filtering procedure, we decided to remove the human element (which correctly was pointed out by several reviewers as non-rigorous and possibly bias-inducing) and implement an automated procedure.

The process now has two steps. First, we initially discarded any single cell time trace that did not have at least 30 time points in each fluorescent channel (the previous value was 10 timepoints). Over the 18 minute window of data acquisition at a time resolution of 15 seconds, this threshold corresponds to roughly half of the time window possessing detectable signal. We reasoned that traces with fewer than 30 time points would have an insufficient amount of data for the inference to work successfully. This reduced the number of cells from 1053 to 427.

Second, instead of manually curating the subsequent data and potentially introducing human bias, we opted for a new methodology that used an automatic cutoff. For each single-cell fit, we calculated the average squared normalized residual δ^2, defined as $\delta^2 = \sum_{timepoints}(F_{data} - F_{fit})^2/F_{data}{}^2$, where the summation occurs over all time points and F_{data} and F_{fit} correspond to the fluorescence data and fit, respectively. Thus, δ^2 gives a measure of how good or bad, on average, each single-cell fit is.

Fig. R6A and B show histograms of the average squared normalized residual δ^2 for the entire n=427 dataset, with log and linear x-axes. We see that the vast majority of data possesses values of δ^2 smaller than unity, with a long tail at higher values corresponding to bad fits. We decided to implement a cutoff of $\delta^2 = 1$, where any cell with a higher value of δ^2 was automatically discarded. This reduced the dataset from 427 cells to 355 cells (in the previous version of the manuscript size, the final dataset size was n=299).

To assess the rejected fits for underlying biological causes, we did a qualitative examination for common features. There were several sources of bad fits. First, some traces possessed low signal-to-noise ratio (Fig. R6C), possibly due to fluctuations in MCP-mCherry or PCP-GFP background fluorescences leading to increased uncertainty, that nevertheless yielded reasonable fits that were slightly above the δ^2 cutoff. Still others simply had poor fits, possibly due to running into issues with the inference algorithm such as getting trapped in local minima (Fig. R6D). We consider improvements to the algorithm to be outside the scope of this work, since the retained data still contain novel, interpretable results.

Finally, one potential biological source confounding the model could be substantial burstiness of the promoter. Although the majority of the traces we analyzed indicated that the *hunchback* reporter gene studied here possessed a promoter that was effectively ON during the cell cycle studied, some traces possessed substantial time dependence of the fluorescence signal, potentially resulting from rapid switching of the promoter between ON and OFF states. From the lens of the model, this would violate the mean-field assumption of the initiation rate term $R(t)$and cause the fluctuations $\delta R$to no longer be small compared to the mean value $< R >$. As seen in a representative example in Fig. R6E, such traces are very time-dependent and are not fit well with the model. Although such bursty behavior is of high biological significance, capturing the behavior would require more specific models (e.g. two-state telegraph models in the flavor of Lammers *et. al.* 2020 *PNAS*), and thus we hope to consider these extensions in future work.

Due to the variety of sources contributing to the rejected fits, we opted for a conservative approach and only analyze the cells with high signal quality that did not exhibit noticeable bursting. The number of retained fits were still much higher than the number of rejected fits (Fig. R6F). Thus, our work provides a self-contained framework applicable for describing the behavior of promoters that are primarily ON for the duration of the experiment.

To check that the curation procedure did not incur substantial bias, we compared the average inferred mean initiation rate, elongation rate, and cleavage time as a function of embryo position between the curated and uncurated datasets (Fig. R6G-I). We observed no substantial difference between the two datasets, indicating that the curation procedure was not systematically altering the inference results.

These details have been included in the updated Section S4.3 and the new Figure S4. We have also expanded the discussion to talk about these bursty traces, starting at Line 520.

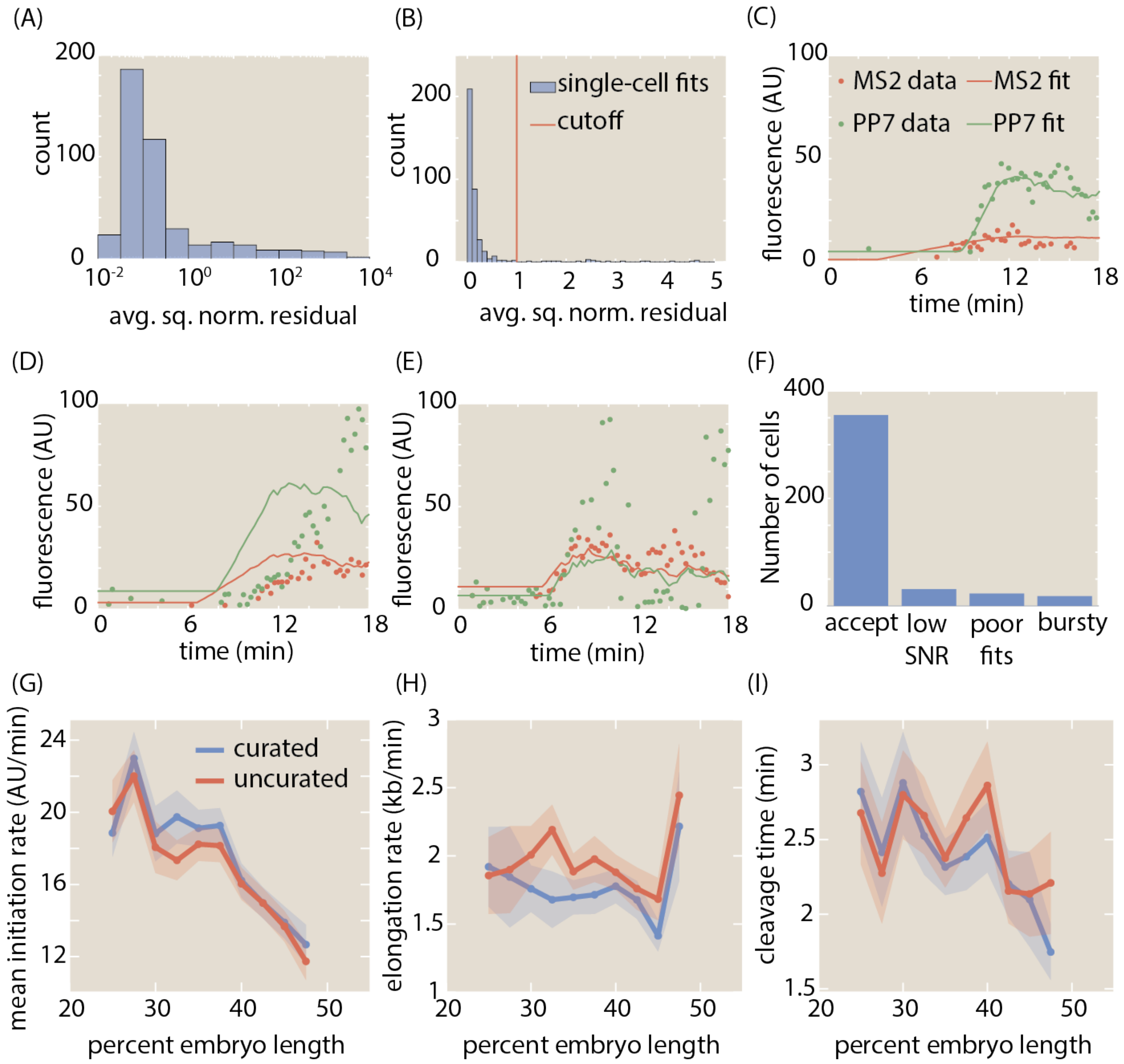

Figure R6 - Automated curation of data. (A, B) Histograms (blue) of average squared normalized residual of single-cell fits, in log (A) and linear (B) scale, with cutoff of $\delta^2 = 1$ shown in red in (B). (C) Example of bad fit from poor signal-to-noise ratio. (D) Example of bad fit of otherwise reasonable data from issues in fitting algorithm, for example due to local minima. (E) Example of bad fit due to potential presence of substantial bursting of promoter. (F) Number of single cell fits in each class of rejected fit, along with number of accepted fits, after the initial filtering based on number of time points. Altogether, 84% of filtered fits were accepted. (G, H, I) Comparison of average inferred (G) mean initiation rate, (H) elongation rate, and (I) cleavage time as a function of embryo position, between curated and uncurated datasets. The data shown in C-E are in each fluorophore's intrinsic arbitrary unit without rescaling, to present the fluorescence intensities in their raw form. Values of δ^2 were 6.05, 1820, and 688 for C-E, respectively. Shading in G-I represents standard error of the mean across 355 and 427 cells across 7 embryos for curated and uncrated datasets, respectively.

13. In section DNA construct, they mentioned the paper Garcia et al.,2013. They used the almost the same DNA construct. However compared with the paper, it showed no background signal inside the cell. More details should be discussed in both DNA construct parts and also behind the figure 2A.

Author response: We are not entirely sure what background signal the reviewer is referring to. Garcia *et al.* used MCP-GFP to label RNA, and a Histone-RFP fusion to label nuclei. The core difference between this work and Garcia *et al.* is that, due to the additional MCP-mCherry channel to acquire MS2 data, a Histone-iRFP fusion was utilized to label nuclei. iRFP has emission and absorption spectra distinct from the GFP and mCherry spectra. The nuclear background is given by the nuclear concentration of free MCP-mCherry and PCP-GFP, which is subtracted when quantifying the fluorescence of the transcription spots. We have updated the text for the "DNA Constructs" methods section as well as the caption for Figure 2A accordingly.

14. In section S7 (line 1130), figure S5C was used to explain the separation of the experimental noise from the biological noise. However the figure showed the fluorescence intensity of MS2/PP7 and a linear fit.

Author response: We have removed this reference to Fig. S5C (now Fig. 3) since it was a bit unclear given the flow of the manuscript.

%%%% Are original data deposited in appropriate repositories and accession/version numbers provided for genes, proteins, mutants, diseases, etc.?

15. In section S4, a construct with alternating MS2/PP7 loops was used to calibrate the signals. The DNA construct is required.

Author response: This DNA construct has been added to the public Benchling folder under the label "pIB-hbP2 p2p-MS2/PP7-48-lacZ-Tub3'UTR".

%%%% Are details of the methodology sufficient to allow the experiments to be reproduced?

16. More details are required in the methods and sample preparation section. In section sample preparation, only the reference papers were mentioned, the whole preparation process should be mentioned too.

Author response: We have added and clarified the sample preparation process in the methods section.

17. In section image analysis, a custom-written software was used to analyze the images. The name and purpose should also be mentioned.

Author response: We have added more details on the image analysis software, and referenced a public GitHub repository containing the codebase.

%%%% Is any software created by the authors freely available?

Github repository

%%%% Minor remarks regarding the modelling part

18. The high RNAP density (Fig. 3 d) seems to contradict the independent particle assumption of the model. Can you clarify why it is legitimate to still use this model?

Author response: We emphasize that our model is meant to produce effective transcriptional parameter values, rather than mechanistically motivated quantities. In particular, while the RNAP densities present in the *hunchback* reporter gene are rather high, little enough is understood about RNAP elongation *in vivo* to be certain of how this high density will affect overall elongation. For example, RNAP molecules in living organisms have been posited to traffic jam and slow down (Klumpp and Hwa, *PNAS*, 2008), or instead push each other and speed up (Galbert, *Biophysical Chemistry*, 2011). Direct observation of either in a live imaging setup has not existed thus far. So, while interactions between RNAP molecules likely exist, we decided to remain flexible and produce an effective model that could produce self-consistent insights when comparing values within the model than to focus on a more mechanistically motivated model (see Line 72 of the manuscript for a clarification of this point).

19. The initiation rate R(t) in the description of the full model in S1 is not fully clear to me. The notation suggests that delta R(t) is a stochastic fluctuation, but from the description in the inference part, it seems like it is treated as a constant offset for each time point.

Author response: The delta R(t) is indeed a constant offset for each time point. We decided to use the delta notation because the mean initiation rate <R> essentially is a mean-field approximation, where the delta R(t) represent small deterministic fluctuations at each time point. We have updated Line 107 of the main text to clarify this.

20. Also, the discussion suggests that the model is in continuous-time. Then, in line 721, a computational time step suddenly appears. This could be explained more explicitly.

Author response: While the model itself can function in continuous time, the computational simulation used for the statistical inference was coded using discrete computational timesteps. We have updated the text in Section S1 to be more clear about this point.

21. From (S4), (S5) it seems that the number of RNAP molecules is a discrete quantity. In contrast, the discussion in S1 around line 720 explains that R(t) dt RNAP molecules are loaded at each time step, which is not an integer.

Author response: Because the simulation relies on discrete numbers of RNAP molecules, after calculating R(t) dt, the subsequent value is rounded down to the nearest integer to be consistent with the discretization. We have updated the text to clarify this.

22. Is there a particular reason mu_x is used in (S16) for the normalization? Why not use x_true as for a standard relative error?

Author response: We used mu_x in the normalization to allow for the direct comparison of overall CV^2 between the simulation and data in Fig. S5. Since both the empirical CV^2 and the squared scaled error defined here have mu_x^2 in the denominator, their magnitudes can be directly compared to gain a sense of how the inference error contributes to the overall noise (Eq. S20). We believe that this presentation makes for ease of visualization and intuition, since scaling Fig. S5 by the empirical values mu_x allows for the interpretation of the inference error in units of the data.

Using x_true for (S16) does not change the conclusions, as seen below in Fig. R7. For the most part, the distributions of both scaled error as defined in the supplement and relative error, defined as (x_infer - x_true)/x_true, lie between -0.5 and 0.5.

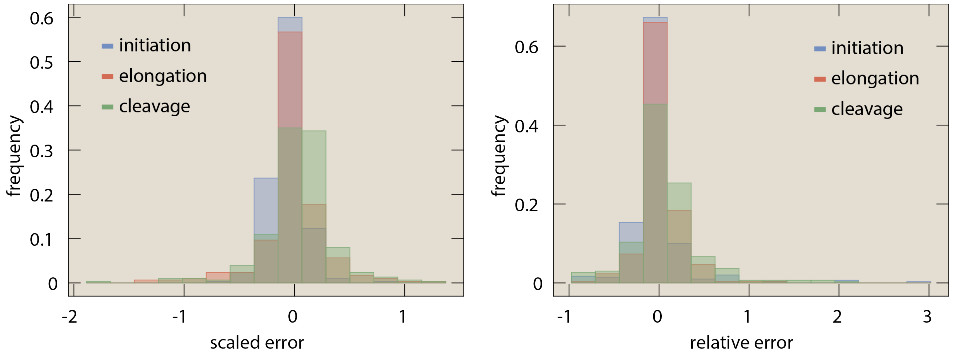

Figure R7 - Comparison of scaled and relative error of inference. (Left) Histogram of scaled errors of transcriptional parameters from simulated data, scaled by dividing by the mean empirical distribution value as defined in the supplement. (Right) Histogram of relative errors of transcriptional parameters from simulated data, scaled by dividing by the single-cell ground truth as standard for relative error. The distributions are similar, indicating that the inference error does not depend strongly on the choice on definition of error.
