# Supplementary material for: Real-time single-cell characterization of the eukaryotic transcription cycle reveals correlations between RNA initiation, elongation, and cleavage
Source: PLoS Comput Biol. 2021 May 18;17(5):e1008999. doi: 10.1371/journal.pcbi.1008999 (PMC8162642; doi:10.1371/journal.pcbi.1008999)
Supplement: Attachment — Submitted filename: TranscriptionCycle_PLoS_ResponseV2.pdf [file pcbi.1008999.s018.pdf]

Reviewer's Responses to Questions

Comments to the Authors:

Please note here if the review is uploaded as an attachment.

We thank the reviewers and reviewing editor for their comments. We've copied these comments below and have responded to them in-line, point-by-point. Where appropriate, we've incorporated these critiques and suggestions as new content into the manuscript, which we believe is much improved with the addition of this feedback. As a result, Figures 5 and S5 have been updated with the new feedback.

We have also fixed typographical errors in supplementary figures S7 and S8, and reformatted supplementary figures S1 and S9 to be consistent with PLoS requirements.

Reviewer #1: In their revised manuscript, the authors have made several improvements through additional analysis and formatting. In particular, the revised work implements an improved observation model with a scaling noise term to relate intensity measurements for different fluorophores. The resulting data more consistently represents the underlying biological phenomena, averting the need for a hierarchical fitting procedure which came across as a 'brute-force' solution in the prior submission. The authors also implement an automated data curation method that removes human bias and preserves a greater number of single-cell trajectories. Overall the revised manuscript seems more substantive and scientifically sound with these changes in combination with wise formatting decisions. Although I still have a couple minor comments, I am satisfied by the author's responses to my concerns in addition to those of my colleagues.

Minor comments:

1. My previous concern #1 related to assumptions of processivity and instantaneous cleavage of mRNA given the observation that mCherry fluorescence intensity can peak and begin decaying while the EGFP channel is still approaching its peak in the same cell. If I understand the response correctly, the argument is that the steady state plateau where mRNA cleavage and production rates are balanced ends earlier for the 5' reporter than the 3' reporter because elongation continues after transcript initiation has ceased. So in this intermediate state there is continued production of the 3' reporter and no new production of the 5' reporter, and the intensity of the 5' reporter will therefore begin decaying because of ongoing cleavage events. Modifications to figure 1D, in

particular the phase marked (vi), clearly indicate this process but I did not find an accompanying explanation in the manuscript or figure legend.

Author response: We thank the reviewer for pointing out our oversight in the description of Fig. 1D. We have added text in the caption to more precisely describe the reasoning in (vi), where the 5’ MS2 signal begins to decrease before the 3’ PP7 signal due to continued elongation after the cessation of initiation.

2. I will push back on the claim “... the computational algorithm itself is quite standard, but the application of Bayesian inference to directly fitting live imaging datasets is novel.” and draw the author’s attention to a couple our recent works PMID: 30175326 and PMID: 32150537. The first of which implements a Bayesian analysis technique to increase global sampling (i.e. preventing capture in local minima) and accelerates convergence for time-course data. The second implements a more advanced Bayesian approach and applies it to live-cell imaging data. These topics may be relevant to the discussion on ‘comparison to existing analysis techniques’ or the ‘future improvements’.

Author response: We agree that these related works merit attention and have expanded the section “Comparison to existing analysis techniques” to include a discussion of these papers.

Reviewer #2: I like to thank the authors for responding to my critique in such detail. After reading the response carefully, I am mostly satisfied with the response of the authors. I disagree with the only point related to statements about the limitations of using RNA-FISH data to model gene expression dynamics from fixed cells. Specifically, the information in lines 454-455 is not correct. RNA-FISH can infer models and rates at a high temporal resolution down to 1min and at similar time scales as done so in this manuscript. What RNA-FISH cannot do is following the same single cell over time. An excellent example of where live cell transcription imaging is gaining novel insight compared to RNA-FISH is understanding mRNA – lncRNA regulation, for example, by work from the Larson lab [1]. The authors' lack of literature review might be their limited knowledge about the work by several groups that successfully used time course snapshot RNA-FISH data of fixed cells to infer rates and mechanisms of transcription similar as done in the current manuscript [2,3,12,13,4–11]. To place the authors' orthogonal work into perspective, I recommend including these and equivalent citations in the document. Specifically, add appropriate sources to the revised manuscripts in lines 96, 259, 319, 423, 455, Sections S5, S8, S10. I believe that live cell and fixed cell experiments of transcription complement each other and demonstrate overlapping conclusions. After this point has been addressed, I recommend the manuscript be published.

Bibliography:

1. Lenstra TL, Coulon A, Chow CC, Larson DR (2015) Single-Molecule Imaging Reveals a Switch between Spurious and Functional ncRNA Transcription. Mol Cell 60: 597–610.

2. Wyart M, Botstein D, Wingreen NS (2010) Evaluating gene expression dynamics using pairwise RNA fish data. PLoS Comput Biol 6: 1000979.

3. Gómez-Schiavon M, Chen LF, West AE, Buchler NE (2017) BayFish: Bayesian inference of transcription dynamics from population snapshots single-molecule RNA FISH in single cells. Genome Biol 18: 164.

4. Lyon K, Aguilera LU, Morisaki T, Munsky B, Stasevich TJ (2019) Live-Cell Single RNA Imaging Reveals Bursts of Translational Frameshifting. Mol Cell 75: 172-183.e9.

5. Xu H, Sepúlveda LA, Figard L, Sokac AM, Golding I (2015) Combining protein and mRNA quantification to decipher transcriptional regulation. Nat Methods 12: 739–742.

6. Neuert G, Munsky B, Tan RZ, Teytelman L, Khammash M, van Oudenaarden A (2013) Systematic Identification of Signal-Activated Stochastic Gene Regulation. Science (80-) 339: 584–587.

7. Munsky B, Li G, Fox ZR, Shepherd DP, Neuert G (2018) Distribution shapes govern the discovery of predictive models for gene regulation. Proc Natl Acad Sci 115: 7533–7538.

8. Miura M, Dey S, Ramanayake S, Singh A, Rueda DS, Bangham CRM (2019) Kinetics of HTLV-1 reactivation from latency quantified by single-molecule RNA FISH and stochastic modelling. PLOS Pathog 15: e1008164.

9. Fei J, Singh D, Zhang Q, Park S, Balasubramanian D, Golding I, Vanderpool CK, Ha T (2015) Determination of in vivo target search kinetics of regulatory noncoding RNA. Science (80-) 347: 1371–1374.

10. So L, Ghosh A, Zong C, Sepúlveda LA, Segev R, Golding I (2011) General properties of transcriptional time series in Escherichia coli. Nat Genet 43: 554–560.

11. Shaffer SM, Dunagin MC, Torborg SR, Torre EA, Emert B, Krepler C, Beqiri M, Sproesser K, Brafford PA, Xiao M, et al. (2017) Rare cell variability and drug-induced reprogramming as a mode of cancer drug resistance. Nature 546: 431–435.

12. Senecal A, Munsky B, Proux F, Ly N, Braye FE, Zimmer C, Mueller F, Darzacq X (2014) Transcription Factors Modulate c-Fos Transcriptional Bursts. Cell Rep 8: 75–83.

13. Albayrak C, Jordi CA, Zechner C, Lin J, Bichsel CA, Khammash M, Tay S (2016) Digital Quantification of Proteins and mRNA in Single Mammalian Cells. Mol Cell 61: 914–924.

Author response: We apologize for our lack of systematic literature review in our discussion of RNA-FISH approaches. Following the reviewer's suggestion, we have incorporated the works mentioned above into our manuscript in the specific places suggested. We agree that live cell imaging and fixed-tissue approaches are very complementary techniques that each present their own set of advantages and disadvantages.

Lines that have been modified in the new manuscript:

Lines 96, 261, 322, 427, 458, 1418, 1452

Reviewer #3: The revised manuscript by Liu et al. has addressed the previous issues with model presentation, data curation and benchmarking of the inference method. The abstract, introduction and discussion have been adjusted to properly reflex the method itself and its applicability, rather than focusing too much on the biological findings. By applying a model of transcription to predict the MS2 traces, with the variance scaling with the mean signal as suggested by reviewer #4, the inferred variance from the data is found to be much higher than the inference error (Fig. S5D). Thus, conclusions on the variations and correlations between parameters are valid.

I only have some comments on this notion of scaled variance. They require very minor discussions but are important. Other than that, I find the revision satisfactory for publication.

-From Fig. S4, the found variance not only scales linearly but is proportional to the mean intensity. This implies that noise emerges purely from the GFP and mCherry molecules bound to nascent RNA, rather than the background noise (i.e. from unbound fluorescent molecules). Normally I would expect a mix of both, especially when using a gaussian filter to extract the MS2/PP7 spot intensity at the detected spot location (as in Garcia et al, 2013, Lucas et al, 2018). In this work, does the calculation of the spot intensity involve such a filter? Or is the spot intensity just the sum of detected spot's pixel intensity, and thus the variance scales with number of detected pixels (i.e. spot size). Please discuss whether the scaling of signal variance can depend on how spot intensity is calculated, which is not always standardized.

Author response: The work here only involves a Gaussian filter to determine the background fluorescence level outside of the detected spot. The MS2/PP7 spot intensity was determined by integrating the overall pixel intensities within a small circular neighborhood around the spot center (with a fixed radius of ~1 micron) and subtracting the corresponding background fluorescence level. While the number of detected pixels does affect the final recorded spot intensity (and thus the variance across measurements), the size of a spot does correlate with the overall transcriptional activity—thus, the scaling of signal variance depends on multiple factors but would be expected to increase with spot brightness, and to a lesser degree, size, both of which contribute to the overall integrated intensity within the neighborhood.

While previous work using this same methodology found that background fluorescence noise was dominant (see e.g. Garcia *et. al*. 2013 Fig. S2E), we speculate that, in our work, the difference in fluorescence noise behavior stems from the use of mCherry

instead of GFP. In our experience, mCherry has a much noisier readout in fruit fly embryos. In addition, other differences such as combining MS2 with mCherry instead of GFP and a different maternal fly line driving different levels of constitutive MCP-mCherry and PCP-GFP could change the relative strength of background fluorescence noise.

We have added this line of reasoning to the end of Section S4.2 at Line 1224.

-Given source of noise in the detected ms2 signal mostly arise from the nascent RNA (noise scaled with loci intensity) rather than from the background intensity (noise unscaled), can you discuss the viability of previous “ensemble” methods, such as memory based HMM or Autocorrelation analysis, which assume only background noise?

Author response: While these other “ensemble” methods assume only background noise, they should still be viable even in the presence of substantial noise in the individual fluorophores themselves, as seen in this work. Because such noise should be uncorrelated, they would manifest as random noise on top of the underlying biological signal. In the case of memory based HMM, the model fitting would likely treat this noise as irrelevant and fit to the mean behavior, unless the noise is high enough where such fitting would be impractical. However, as seen in, for example, Lammers et al 2020 (PNAS), HMM models can successfully extract insights from these sorts of live imaging data.

Autocorrelation should also work fine since it typically involves averaging of many single cell traces, and we would expect the contributions from fluorescence noise to average out after enough statistical samples.

-Please use p-val or p-value instead of p, since it is confusing when placed next to rho sign.

Author response: We have changed the text to say “p-val” instead of “p,” as well as the text inside the Fig. 5 in the main text and Fig. S5 in the supplement.

-Line 101. Closing bracket needed

Author response: We have added the missing parenthesis.

-Line 986. Please continue from “…”

Author response: We have finished the missing text in the sentence, which now says "As a result, R(t) dt is rounded down to the nearest integer since the model cannot load fractional numbers of RNAP molecules."
